# A New Platinum-Based Prodrug Candidate for Chemotherapy and Its Synergistic Effect With Hadrontherapy: Novel Strategy to Treat Glioblastoma

**DOI:** 10.3389/fnins.2021.589906

**Published:** 2021-03-22

**Authors:** Beatrice Ferrari, Elisa Roda, Erica Cecilia Priori, Fabrizio De Luca, Angelica Facoetti, Mauro Ravera, Federico Brandalise, Carlo Alessandro Locatelli, Paola Rossi, Maria Grazia Bottone

**Affiliations:** ^1^Department of Biology and Biotechnology “L. Spallanzani”, University of Pavia, Pavia, Italy; ^2^Laboratory of Clinical & Experimental Toxicology, Pavia Poison Centre, National Toxicology Information Centre, Toxicology Unit, Istituti Clinici Scientifici Maugeri IRCCS, Pavia, Italy; ^3^National Center of Oncological Hadrontherapy (Fondazione CNAO), Pavia, Italy; ^4^Department of Sciences and Technological Innovation (DiSIT), University of Piemonte Orientale “A. Avogadro”, Alessandria, Italy; ^5^Department of Fundamental Neurosciences (NEUFO), University of Geneva, Geneva, Switzerland

**Keywords:** platinum(IV) chemotherapeutics, hadrontherapy, carbon ions irradiation effects, tumor resistance, glioblastoma (GBM), glioblastoma, *in vitro*

## Abstract

Glioblastoma (GBM) is the most common tumor of the central nervous system. Current therapies, often associated with severe side effects, are inefficacious to contrast the GBM relapsing forms. In trying to overcome these drawbacks, (*OC*-6-44)-acetatodiamminedichlorido(2-(2-propynyl)octanoato)platinum(IV), also called Pt(IV)Ac-POA, has been recently synthesized. This new prodrug bearing as axial ligand (2-propynyl)octanoic acid (POA), a histone deacetylase inhibitor, has a higher activity due to (i) its high cellular accumulation by virtue of its high lipophilicity and (ii) the inhibition of histone deacetylase, which leads to the increased exposure of nuclear DNA, permitting higher platination and promoting cancer cell death. In the present study, we investigated the effects induced by Pt(IV)Ac-POA and its potential antitumor activity in human U251 glioblastoma cell line using a battery of complementary techniques, i.e., flow cytometry, immunocytochemistry, TEM, and Western blotting analyses. In addition, the synergistic effect of Pt(IV)Ac-POA associated with the innovative oncological hadrontherapy with carbon ions was investigated, with the aim to identify the most efficient anticancer treatment combination. Our *in vitro* data demonstrated that Pt(IV)Ac-POA is able to induce cell death, through different pathways, at concentrations lower than those tested for other platinum analogs. In particular, an enduring Pt(IV)Ac-POA antitumor effect, persisting in long-term treatment, was demonstrated. Interestingly, this effect was further amplified by the combined exposure to carbon ion radiation. In conclusion, Pt(IV)Ac-POA represents a promising prodrug to be incorporated into the treatment regimen for GBM. Moreover, the synergistic efficacy of the combined protocol using chemotherapeutic Pt(IV)Ac-POA followed by carbon ion radiation may represent a promising approach, which may overcome some typical limitations of conventional therapeutic protocols for GBM treatment.

## Introduction

Glioma is a broad category of glial brain and spinal cord tumors which originate in the glial cells that surround and support neurons in the brain, including astrocytes, oligodendrocytes, and ependymal cells. About 33% of all brain tumors are gliomas, accounting for about 80% of the total malignant central nervous system (CNS) tumors in adults (Hanif et al., 2017).

Among these, glioblastoma (GBM) is one of the most common and aggressive primary brain tumor (Davis, 2016), characterized by diffuse infiltration of the adjacent brain parenchyma and development of drug resistance to standard treatment (Chen et al., 2018). So far, GBM remains associated with an extremely aggressive clinical course, and only 0.05–4.7% of patients survive 5 years from diagnosis (Ostrom et al., 2018).

Cellular pleomorphism with nuclear atypia, high mitotic activity, and microvascular proliferation distinguish GBM from other lower-grade gliomas (Hambardzumyan and Bergers, 2015). In addition, the inter- and intra-patient tumor heterogeneity causes several obstacles, limiting the improvement of an early diagnosis and treatment protocols. The tumor microenvironment (TME) plays a crucial role in mediating tumor progression and invasiveness, contributing to tumor aggression and poor prognosis (Yekula et al., 2020). Recent studies have shown that differentiated tumor cells may have the ability to dedifferentiate acquiring a stem-like phenotype in response to microenvironment stresses such as hypoxia. Acidic extracellular pH and nitric oxide were also shown to be involved in stemness preservation (Dahan et al., 2014).

Currently, the standard of care consists of surgical resection followed by radiotherapy (RT) and concomitant and adjuvant chemotherapy. Despite this aggressive treatment regimen, the median survival is only around 15 months, and the 2-year survival rate is only 26.5% (von Neubeck et al., 2015; Chen et al., 2018). Indeed, due to the location of GBM origin and its infiltrative growth (Urbańska et al., 2014), complete surgical resection of the tumor is often not possible other than with a high risk of neurological damages for the patient (Goldbrunner et al., 2018). The main chemotherapeutic agent employed for GBM treatment is temozolomide (TMZ) (Strobel et al., 2019); however, intrinsic or acquired resistance to TMZ often defines the poor efficacy of this drug. Notably, the expression of *O*^6^-methylguanine-DNA methyl-transferase (MGMT) unmethylated promoter in some patients is one of the principal mechanisms responsible for this chemoresistance, reducing mean patients’ survival (Chen et al., 2018).

Cisplatin (*cis*-dichlorodiammineplatinum, CDDP) is another alkylating agent used as an anticancer chemotherapy drug employed to treat various types of malignancies, including GBM (Dasari and Tchounwou, 2014; Pérez et al., 2019). Despite initial benefits, CDDP is often associated with severe systemic toxicity, which occurs especially after long-term treatment (Karasawa and Steyger, 2015; Chovanec et al., 2017). Among these adverse side effects, neurotoxicity assumed increasing clinical importance as it is dose-cumulative and becomes limiting in long-lasting therapies, thus decreasing CDDP’s clinical use in GBM treatment (Staff et al., 2019).

In the attempt to circumvent all the above reported drawbacks, a large number of “non-classical” platinum complexes have been prepared and tested for anticancer activity. In this view, the synthesis of new platinum(IV)-based prodrugs, Pt(IV) may pose great advantages allowing the addition of one or two adjuvant/synergistic agents to the parent cytotoxic Pt(II) square-plane scaffold in axial position. The axial ligands can be used to improve the physical and chemical properties of the complex, designing multifunctional prodrugs, often called “combo” (Gabano et al., 2014; Kenny et al., 2017; Sabbatini et al., 2019). In this way, they can improve the lipophilicity and, consequently, influence passive diffusion, promoting a synergistic intracellular accumulation (Ravera et al., 2015; Raveendran et al., 2016).

In addition, these Pt(IV) derivatives, acting as prodrugs, are rather inert to ligand substitution or hydrolysis, minimizing the off-target interactions and the side effects on healthy cells typical of the more reactive Pt(II) progenitors. Pt(IV) prodrugs can then be reduced in the hypoxic intracellular milieu of tumor cells to the resultant cytotoxic Pt(II) metabolites with the simultaneous loss of the adjuvant or synergistic agents from the axial positions (Najjar et al., 2017; Sabbatini et al., 2019).

In this perspective, the innovative complex (*OC*-6-44)-acetatodiamminedichlorido(2-(2-propynyl)octanoato) platinum(IV), named Pt(IV)Ac-POA, has been recently synthesized starting from the CDDP which is oxidized with hydrogen peroxide to obtain an intermediate octahedral compound, which is then esterified with the addition of two long-chain carboxylic acids. Pt(IV)Ac-POA contains a different medium-chain fatty acid–histone deacetylase inhibitor (MCFA-HDACi), namely 2-(2-propynyl)octanoate (POA), along with an inert acetate (Ac) as axial ligands (Gabano et al., 2017). This prodrug, bearing as axial ligand POA, has a higher activity because of the high cellular accumulation due to its high lipophilicity and to the inhibition of histone deacetylase that leads to the increased exposure of nuclear DNA, thereby permitting higher platination levels at DNA and promoting cancer cell death (Gabano et al., 2017; Novohradsky et al., 2017). Indeed, the HDACi activity of free POA has been established as an inducer of a strong histone H3 acetylation (at lysine 9 level), presumably acting at the HDAC8 level, contributing to increasing the overall anti-proliferative activity (Oehme et al., 2009; Gabano et al., 2017; Sabbatini et al., 2019). Some experimental studies demonstrated that the new prodrug Pt(IV)Ac-POA exhibited promising antitumor activity both *in vitro*, on different human tumor cell lines, as well as *in vivo*, already at concentrations lower than those standardly used for CDDP, also supporting a reduction of undesirable cytotoxicity on healthy tissues (Gabano et al., 2017; Rangone et al., 2018; Ferrari et al., 2020).

Combined with conventional and innovative chemotherapeutic protocols, radiotherapy has always played an important role in the treatment of deep-seated tumors (Liauw et al., 2013; Gupta et al., 2020). However, it has non-eliminable weak points that derive from the physical nature of the photons, being unable to be completely focused on tumor mass, also damaging healthy tissues adjacent to the target, and so increasing the risk of detrimental damage. In the past decades, endless progress of hadrontherapy (HT) occurred, bringing technical innovations both in clinical and scientific research (Rossi, 2015; Marvaso et al., 2017). Hadrontherapy displayed less invasiveness than conventional radiotherapy, being also more effective compared to X-ray radiotherapy (Combs et al., 2013). While megavoltage photons used in conventional radiotherapy deposit energy uniformly through the tissue, with the exception of a buildup region in superficial tissue, where a relative dose-sparing occurs (Tinganelli and Durante, 2020), protons and carbon ions, releasing energy at the inverse of their velocity, present a pronounced peak, called the Bragg peak, at a deeper point (Ebner and Kamada, 2016). Indeed, carbon ion beams allow an improved dose distribution, leading to the concentration of enough dose within a target volume while minimizing the dose in the adjacent healthy tissues (Kamada et al., 2015). Furthermore, in contrast to X-rays, protons and ions are heavy particles, so they can penetrate the tissue without deviating much from the initial direction, and with their electric charge, they tear electrons from the tissue molecules, depositing most of their energy in the last centimeters of the path, providing a higher action (Kamada et al., 2015) and, in the case of carbon ions, achieving a greater relative biological effectiveness (RBE). Carbon ions are also less dependent on the oxygen enhancement ratio (OER), which in radiobiology refers to the increased effect of ionizing radiation due to the presence of oxygen. This would allow carbon ion radiotherapy (CIRT) to eradicate hypoxic glioblastoma cells, for example following an anti-angiogenic therapy. The induction of apoptosis, autophagy, and cellular senescence is a set of mechanisms underlying the killing of glioblastoma cells mediated by the irradiation of carbon ions (Jinno-Oue et al., 2010; Tomiyama et al., 2010). Furthermore, it has been demonstrated that such radiations would be able to inhibit the migratory capacity of glioma cells through a reduction in the expression of integrins (Rieken et al., 2012). It has also recently been hypothesized that carbon ion radiation can overcome the intrinsic radioresistance of cancer stem cells (Pignalosa and Durante, 2012); besides, it may be a promising therapy for pediatric brain tumors, decreasing side effects related to CNS sensibility (Laprie et al., 2015; Combs, 2018).

Based on all these previously reported knowledge, the present work aimed at characterizing *in vitro* the action of the recently synthesized platinum-based compound Pt(IV)Ac-POA using human U251 cell lineage as one of the GBM-specific models, typically characterized by drug resistance properties (Naidu et al., 2010; Liu et al., 2015; Lin et al., 2018). Specifically, a first experimental phase was conducted to identify the Pt(IV)Ac-POA efficient cytotoxic concentration and the involved cell death pathways after both short- and long-term exposure, also assessing the potential clonogenicity impairment. Then, the second step of the study was devoted to addressing the potential synergistic action of Pt(IV)Ac-POA treatment followed by carbon ion radiation, with the final goal of assessing the efficacy and feasibility of a therapeutic protocol combining chemotherapy and carbon ion radiation therapy in acute and long-term GBM treatment.

## Materials and Methods

### Cell Culture

Human U251 MG cell line (Sigma-Aldrich, Milan, Italy) was cultured in Eagle’s minimal essential medium (EMEM) supplemented with 2 mM l-glutamine, 1% non-essential amino acid (NEAA), 1% sodium pyruvate, 10% fetal bovine serum (FBS), 50 IU/ml penicillin, and 50 μg/ml streptomycin. The cell culture was maintained at 37°C in a humidified atmosphere (95% air/5% CO_2_). All cell culture reagents were purchased from Celbio S.p.a. and Euroclone S.p.a. (Pero, Milan, Italy).

### Experimental Design

#### Pt(IV)Ac-POA Dose Selection: Cell Viability and Proliferation Evaluated by MTS Assay

In order to select the proper Pt(IV)Ac-POA dose to be used in all the following analyses, as a first experimental step, a range of Pt(IV)Ac-POA concentrations was evaluated through the MTS [3-(4,5-dimethylthiazol-2-yl)-5-(3-carboxymethoxyphenyl)-2-(4-sulfophenyl)-2H-tetrazolium] assay.

Briefly, the cell viability test was performed using the CellTiter 96^®^ AQueous One Solution Cell Proliferation Assay (Promega) kit. A volume of 200 μl of cells was suspended at a density of 5,000 cells/well, transferred to a 96-well plate (0.2 ml per well), and incubated at 37°C for 24 h in a humidified atmosphere containing 5% of CO_2_. Subsequently, the culture medium was replaced with a fresh medium to then carry out the requested treatment. As a control, the cells were incubated with the culture medium alone. For Pt(IV)Ac-POA exposure, a range of concentrations, ranging from 1 to 40 μM, was prepared by dissolving the prodrug in the specific culture medium. The dose range was chosen based on previous works in which the effects of the prodrug Pt(IV)Ac-POA were assessed on tumor cell lines of the nervous system, i.e., B50 neuroblastoma and C6 glioma rat cells, respectively (Rangone et al., 2018; Ferrari et al., 2020). Forty-eight hours after exposure, the culture medium was replaced with fresh medium, the MTS solution (20 μl/well) was added to each well in the darkness, and the plates were then incubated for about 3 h at 37°C. At the end of the incubation time, the quantification was performed by measuring the samples’ absorbance at 490 nm with the ELx808TM Absorbance Microplate Reader (Bio-Tek Instruments, Inc.). Data were expressed as a percentage of control.

Percentage cell viability was calculated using the following formula:

Cellviability(%)=(Abs490treatedcells/Abs490controlcells)×100

#### Pharmacological Treatment and Exposure Conditions

Forty-eight hours before the experiments, the cells were seeded on glass coverslips (20,000 cells) for fluorescence microscopy evaluations or grown in 75-cm^2^ plastic flasks for flow cytometry, Western blotting, and transmission electron microscope (TEM) ultrastructural analysis. Cell exposure to chemotherapeutic drugs was performed at 37°C. To compare the efficacy of the prodrug Pt(IV)Ac-POA to that observed after the conventional CDDP treatment (Teva Pharma, Milan, Italy), a 40 μM CDDP concentration was selected based on previous *in vitro* investigations (Bottone et al., 2008; Grimaldi et al., 2019) as well as *in vivo* experimental studies (Bottone et al., 2008; Cerri et al., 2011), employing a single subcutaneous injection (5 μg/g, b.w.) in 10-day-old rats, corresponding to the therapeutic dose already employed in clinical practice (Bodenner et al., 1986; Dietrich et al., 2006).

Cell lines were exposed to the diverse platinum compounds according to the following protocols:

(i) Standard acute test: 48-h continuous treatment (CT) to Pt(IV)Ac-POA or CDDP.

(ii) Standard acute test (48-h CT) to Pt(IV)Ac-POA or CDDP, followed by a 7-day recovery phase in drug-free normal EMEM, namely “recovered” (REC) condition.

#### Carbon Ion Radiotherapy

**FIGURE 1:**
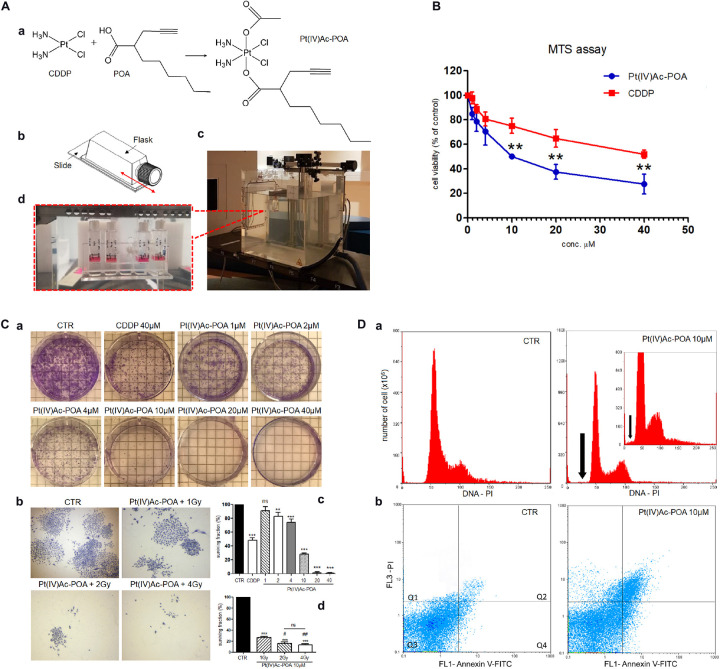
**(A)** Schematic representation of (*OC*-6-44)-acetatodiamminedichlorido (2-(2-propynyl)octanoato)platinum(IV) [Pt(IV)Ac-POA] synthesis process and carbon ion irradiation. **(a)** Cisplatin (*cis*-dichlorodiammineplatinum, CDDP), 2-(2-propynyl)octanoic acid (POA), and the related Pt(IV) mixed derivative (Pt(IV)Ac-POA). **(b)** Flask sterile on slide used for U251 cell culture irradiation. **(c)** Equipment setup in the irradiation room facility at the CNAO Foundation. **(d)** Flasks positioned in a water phantom placed at a depth of 15 cm, corresponding to the central position of a 6-cm-wide spread-out Bragg peak (SOBP), even before irradiation with a horizontal beam. **(B)** Effects of Pt(IV)Ac-POA and CDDP on cell viability and proliferation of human U251 MB cell lineage. Cell viability/proliferation obtained using MTS assay after standard acute exposure, i.e., 48-h continuous treatment (CT), to increasing concentrations (1–40 μM) of either Pt(IV)Ac-POA or CDDP. The relative cell viability is expressed as a percentage relative to the untreated control cells. Data represent the mean ± SD. Statistical analysis by Student’s *t*-test: different from CDDP (^∗∗^*p* < 0.01). **(C)** Clonogenic cell survival assay showing the dose–response effect after 48-h CT to increasing concentrations of Pt(IV)Ac-POA alone **(a,c)** and combined with increasing carbon ion radiation doses (1–4 Gy) **(b,d)**. **(c,d)** Histograms showing the U251 surviving fraction (in percent). Statistical analysis by one-way ANOVA followed by Dunnett’s test (histograms in **(c)** or *post hoc* Bonferroni’s test (histograms in **d**). Statistical significance calculated as follows: ^∗^control *vs*. each experimental condition; ^#^ Pt(IV)Ac-POA + 1 Gy *vs*. Pt(IV)Ac-POA CDDP + 2 or 4 Gy; *ns*, not significant. ^∗^*p* < 0.05; ^∗∗^*p* < 0.01; ^∗∗∗^*p* < 0.001; ^#^*p* < 0.05; ^##^*p* < 0.01; ns: not significant. **(D)** Flow cytometry data after annexin V and propidium iodide (PI) staining. **(a)** Cytograms showing the DNA content after PI staining in U251 MB cell lineage: controls *vs*. treated cells [10 μM Pt(IV)Ac-POA 48-h CT]. *Insert* shows the cytogram of the 10 μM Pt(IV)Ac-POA-treated sample compared to a control sample with an identical number of analyzed cells (*y* = 800 × 10^6^). The *black arrow* indicates the sub-G_1_ peak corresponding to dead cells. **(b)** Dual-parameter cytograms of FITC-labeled annexin V (FL1) *vs*. PI staining (FL3) in controls (CTR) and cells exposed to 10 μM Pt(IV)Ac-POA 48-h CT. Quadrants Q1, Q2, Q3, and Q4 show necrotic, late apoptotic, viable, and early apoptotic cells, respectively.

Before the experiments, U251 cells were seeded on culture flasks or flasks sterile on slide 18 × 50 mm (200,000 cells) (Thermo Scientific^TM^ Nunc^TM^ Lab-Tek^TM^) for fluorescence microscopy (Figure 1Ab). Then, the cells were treated with platinum compounds for 48-h CT. At the end of this drug exposure, U251 cells were irradiated with the clinical carbon ion beam at the CNAO Foundation in Pavia. In detail, the flasks were positioned in a water phantom placed at a depth of 15 cm, corresponding to the central position of a 6-cm-wide (from 12 to 18 cm depth in water) spread-out Bragg peak (SOBP) (Figures 1Ac,d). The cells were vertically irradiated with a horizontal beam according to the protocol envisaged for clinical use of carbon ion therapy at CNAO (Facoetti et al., 2015; Mirandola et al., 2015). The SOBP, homogeneous in terms of the dose absorbed in water, was obtained with a modulation of the pencil beam using 31 different energies in the range from 246 to 312 MeV/u. The linear energy transfer (LET) at a depth of 15 cm is equivalent to about 46 keV/u. For this study, the cellular samples were irradiated at room temperature with 2 or 4 Gy physical dose. The irradiation times varied between 15 and 30 min to reach a dose of 2 or 4 Gy, respectively. These radiation doses were selected based on previous literature data considering treatments carried out *in vitro* and related to the fractionated focal irradiation in daily fractions of 2 Gy given 5 days per week for 6 weeks, for a total of 60 Gy used in GBM patients and other tumors (Stupp et al., 2005; Dhermain, 2014; Roach et al., 2018; Ma et al., 2019).

At the end of the experiment, for the analysis of the “standard acute test” condition, pellets for protein extraction were immediately obtained from flasks, while the cells of the flasks sterile on slide were fixed with 4% formalin for 20 min and post-fixed with 70% ethanol at −20°C for at least 24 h for immunocytochemical procedures. In parallel, to evaluate the “REC” condition, at the end of the irradiation, the medium was discarded and replaced with a drug-free fresh medium, followed by a 7-day recovery phase, and then the samples were collected for protein extraction and immunohistochemical procedures as described above.

#### Clonogenic Cell Survival Assay

Clonogenic assay was performed as previously reported (Shen et al., 2015; Ballestreri et al., 2018; Riaz et al., 2019). Briefly, U251 cells were treated as indicated [40 μM CDDP or 1–40 μM Pt(IV)Ac-POA] for 48-h CT. Next, they were removed using trypsin–EDTA 0.10%, seeded in six-well plates, and incubated for 10 days undisturbed, allowing them to form colonies in drug-free medium. To investigate the effects of 10 μM Pt(IV)Ac-POA plus carbon ion radiation, the cells were irradiated with 1, 2, or 4 Gy physical dose after exposure to Pt(IV)Ac-POA (10 μM). The cells were then plated in six-well plates at low densities and colonies were counted after 10 days.

After this 10-day time window, the colonies were gently washed with phosphate-buffered saline (PBS), fixed, and stained with crystal violet solution (0.5% in H_2_O/methanol, 1:1) (Sigma Chemical Co., St. Louis, MO, United States). Stained colonies, defined as groups of ≥ 50 cells, were scored using an Olympus CKX41 inverted microscope combined with an Olympus MagniFire digital camera. Plating efficiency (PE) was calculated as the number of colonies counted divided by the number of cells seeded, considering the colonies formed by control cells as 100%. The average of these values was reported as “surviving fraction.”

### Flow Cytometry

After 48-h CT to the diverse chemotherapeutics, the cells were detached by mild trypsinization (0.25% in PBS, with 0.05% EDTA) to obtain single-cell suspensions to be processed for flow cytometry with a Partec PAS-III flow cytometer (Münster, Germany), equipped with argon laser excitation (power, 200 mW) at 488 nm. Data were analyzed with the built-in software (Flowmax, Partec).

#### Cell Cycle Analysis

The cells were washed in PBS, permeabilized in 70% ethanol for 10 min, treated with RNase A 100 U/ml, and then stained for 10 min at room temperature (RT) with propidium iodide (PI) 50 μg/ml (Sigma-Aldrich, Milan, Italy) 1 h before flow cytometry analysis. PI red fluorescence was detected with a 610-nm long-pass emission filter. At least 20,000 cells per sample were measured to obtain the distribution among the different phases of the cell cycle and the percentage of apoptotic cells.

#### Annexin V Assay: Apoptosis Identification

Single-cell suspensions, obtained as described above, were incubated with annexin V–fluorescein isothiocyanate (FITC) (Annexin V-FITC Apoptosis Detection Kit, Abcam, Italy) for 10 min in the dark at RT. PI was used as a counterstain to discriminate necrotic/dead cells from apoptotic ones. Fluorescence was revealed by means of flow cytometry at 488 nm excitation and with 530/30 (FITC) and 585/42 nm (PI) band-pass emission filters.

### TEM Ultrastructural Investigation

Control and treated cells were harvested by mild trypsinization (0.25% trypsin in PBS containing 0.05% EDTA) and collected by centrifugation at 800 rpm for 5 min in fresh tubes. The samples were immediately fixed with 2.5% glutaraldehyde (Polysciences, Inc., Warrington, PA, United States) in culture medium (2 h at room temperature), centrifuged at 2,000 rpm for 10 min, and washed in PBS. Later, the samples were post-fixed in 1% OsO_4_ (Sigma Chemical Co., St. Louis, MO, United States) for 2 h at room temperature and washed in water. The cell pellets were pre-embedded in 2% agar, and dehydrated with increasing concentrations of acetone (30, 50, 70, 90, and 100%, respectively). Finally, the pellets were embedded in Epon resin and polymerized at 60°C for 48 h. Ultrathin sections were obtained with ultramicrotome Rechter, then located on nickel grids, and stained with uranyl acetate and lead citrate. Sections were observed under a Zeiss EM 900 TEM (Carl Zeiss S.p.A., Milan, Italy) operating at 80 kV. The plates, after being developed, have been computerized through an Epson Perfection 4990 photo scanner at a resolution of 800 dpi and then processed using the Epson Scan software.

### Western Blotting

Control and treated cells were washed twice with PBS and lysed in RIPA (radioimmunoprecipitation assay) buffer (1 M Tris-HCl, pH 7.6, 0.5 M EDTA, pH 8, 5 M NaCl, 100% NP40 Nonidet), with the addition of protease and phosphatase inhibitors at 4°C for 30 min. Proteins were quantified using the Bradford reagent (Sigma-Aldrich, Milan, Italy). Samples were electrophoresed in a 15% SDS-PAGE minigel and transferred onto a nitrocellulose membrane (BioRad, Hercules, CA, United States) by semidry blotting for 1.30 h under a constant current of 60 mA. The membranes were saturated for 30 min with PBS containing 0.2% Tween–20 and 5% skim milk and incubated overnight with the antibody reported in Table 1. After several washes with PBS-Tween, the membranes were incubated for 30 min with the proper secondary antibody conjugated with horseradish peroxidase. Immunoreactive bands were detected with the reagent Luminata^TM^ Crescendo (Merk Millipore, Billerica, MA, United States), according to the appropriate instructions, and revealed on Amersham HyperfilmTM ECL (GE Healthcare, Little Chalfont, United Kingdom) slabs. ImageJ software was used to obtain the density bar chart of the protein bands which are normalized to the respective actin and the loading control. At least three independent experiments were carried out.

**TABLE 1:** Antibodies employed for Western blotting analyses.

**Antigen**	**Primary antibody**	**Dilution in PBS**	**Secondary antibody**	**Dilution in PBS**
*PARP-1*	Rabbit monoclonal anti-PARP-1 (Cell Signaling Technology, Danvers, United States)	1:1000	Goat anti-rabbit horseradish peroxidase (Dako, Milan, Italy)	1:2000
*p62/SQSTM1*	Mouse monoclonal anti- p62/SQSTM1 (Abcam, Cambridge, United States)	1:1000	Goat anti-mouse horseradish peroxidase (Dako, Milan, Italy)	1:2000
*Actin*	Mouse monoclonal anti-actin (Developmental Studies Hybridoma Bank, Iowa City, United States)	3:500	Goat anti-mouse horseradish peroxidase (Dako, Milan, Italy)	1:2000

### Immunofluorescence Reactions

Control and treated cells were grown on coverslips, fixed with 4% formalin (20 min), and post-fixed with 70% ethanol at −20°C for at least 24 h. The samples were rehydrated for 10 min in PBS and then immunolabeled with primary antibodies diluted in PBS for 1 h at RT in a dark moist chamber. The cells were then washed three times with PBS and incubated for 45 min with the proper secondary antibody diluted in PBS. The cells were therefore counterstained for DNA with 0.1 μg/ml of Hoechst 33258 (Sigma-Aldrich, Milan, Italy) for 6 min, washed with PBS, and finally mounted in a drop of Mowiol (Calbiochem-Inalco, Italy) for fluorescence microscopy. For each experimental condition, three independent experiments were carried out.

An Olympus BX51 microscope equipped with a 100-W mercury lamp was used under the following conditions: 330–385 nm excitation filter (excf), 400 nm dichroic mirror (dm), and 420 nm barrier filter (bf) for Hoechst 33258; 450–480 nm excf, 500 nm dm, and 515 nm bf for the fluorescence of Alexa 488; 540 nm excf, 580 nm dm, and 620 nm bf for Alexa 594. Images were recorded with an Olympus MagniFire camera system and processed with the Olympus Cell F software. The primary and secondary antibodies used for immunofluorescence reactions are summarized in Table 2.

**TABLE 2:** Primary/secondary antibodies and respective dilution used for immunofluorescence experimental procedures.

**Antigen**	**Primary antibody**	**Dilution in PBS**	**Secondary antibody**	**Dilution in PBS**
*Caspase-3*	Rabbit monoclonal anti-caspase-3 (Cell Signaling Technology, Danvers, United States)	1:200	Alexa 594-conjugated anti-rabbit antibody (Alexa Fluor, Molecular Probes, Invitrogen)	1:200
*Caspase-8*	Rabbit monoclonal anti-caspase-8 (Cell Signaling Technology, Danvers, United States)	1:100	Alexa 594-conjugated anti-rabbit antibody (Alexa Fluor, Molecular Probes, Invitrogen)	1:200
*PARP-1*	Rabbit monoclonal anti-PARP-1 (Cell Signaling Technology, Danvers, United States)	1:200	Alexa 594-conjugated anti-rabbit antibody (Alexa Fluor, Molecular Probes, Invitrogen)	1:200
*RIP1*	Rabbit polyclonal anti-RIP1 (Santa Cruz Biotechnology)	1:200	Alexa 594-conjugated anti-rabbit antibody (Alexa Fluor, Molecular Probes, Invitrogen)	1:200
*MLKL*	Mouse monoclonal Anti-MLKL Antibody, clone 3H1 (Sigma-Aldrich)	1:200	Alexa 594-conjugated anti-mouse antibody (Alexa Fluor, Molecular Probes, Invitrogen)	1:200
*LC3B*	Rabbit polyclonal anti-LC3B (Cell Signaling Technology, Danvers, United States)	1:400	Alexa 594-conjugated anti-rabbit antibody (Alexa Fluor, Molecular Probes, Invitrogen)	1:200
*p62/SQSTM1*	Mouse monoclonal anti- p62/SQSTM1 (Abcam, Cambridge, United States)	1:100	Alexa 488-conjugated anti-mouse antibody (Alexa Fluor, Molecular Probes, Invitrogen)	1:200
*AIF*	Rabbit polyclonal anti-AIF (Cell Signaling Technology, Danvers, United States)	1:200	Alexa 594-conjugated anti-rabbit antibody (Alexa Fluor, Molecular Probes, Invitrogen)	1:200
*Golgi*	Human autoimmune serum recognizing proteins of Golgi Apparatus^a^	1:200	Alexa 594-conjugated anti-human antibody (Alexa Fluor, Molecular Probes, Invitrogen)	1:200
*Mitochondria*	Human autoimmune serum recognizing the 70 kDa E2 subunit of the pyruvate dehydrogenase complex^b^	1:200	Alexa 594-conjugated anti-human antibody (Alexa Fluor, Molecular Probes, Invitrogen)	1:200
*α-tubulin*	Mouse monoclonal anti-α-tubulin (Cell Signaling Technology, Danvers, United States)	1:1000	Alexa 488-conjugated anti-mouse antibody (Alexa Fluor, Molecular Probes, Invitrogen)	1:200
*Actin*	Alexa 488-Phalloidin/Alexa 594-Phalloidin (Molecular Probes, Invitrogen)	1:500	—	—

*^*a*^Santin et al., 2011; ^*b*^Bottone et al., 2008.*

#### Immunocytochemical Evaluations

For the immunofluorescence quantifications, three independent experiments were performed for each experimental condition. After reactions, image acquisition was performed by Cell F software and the analysis was achieved using ImageJ software. For each condition, 11 quadrants were evaluated for a random analysis. The channels of each fluorescence have been split to obtain the single images in a grayscale, where the minimum value is 0 (black) and the maximum value is 255 (white). Then, the mean values were normalized to the control and expressed as a percentage.

### Statistical Analysis

In the present study, data are presented as the mean ± SEM or mean ± SD over the mean experimental values of each of three independent experiments. The statistical analyses were carried out using Student’s *t*-test or one-way ANOVA, followed by either *post hoc* Bonferroni’s test or Dunnett’s test, performed using GraphPad Prism Inc. 5.03 (GraphPad Software Inc., La Jolla, CA, United States) and R software. A *ρ* value < 0.05 was considered statistically significant.

## Results

The Pt(IV)Ac-POA concentration range presently tested on U251 cells was chosen based on previous *in vitro* data obtained with conventional CDDP (40 μM) and new platinum(II) compounds such as [Pt(*O*,*O*′-acac)(γ-acac)(DMS)]. The MTS assay data revealed that both CDDP and Pt(IV)Ac-POA (Figures 1A,B) caused a dose-dependent cytotoxicity, with the prodrug showing a significant more marked effect already at 10 μM, which became more pronounced at the higher doses, i.e., 20 and 40 μM. In particular, the Pt(IV)Ac-POA concentration of 10 μM was found to be a half maximal inhibitory concentration, able to induce cell death, causing a significant decrease (about 50%) in the number of living proliferating cells (Figure 1B). In addition, the clonogenic assay results revealed that (i) 40 μM CDDP was able to cause a 50% colony formation reduction, while (ii) exposure to increasing Pt(IV)Ac-POA concentrations induced a dose-dependent colony formation inhibition, with a surviving fraction of 30% after the 10 μM dose and a further clonogenic impairment (>90%) at the higher tested doses (20 and 40 μM) (Figures 1Ca,c).

Based on these results, the concentration of 10 μM Pt(IV)Ac-POA was selected, under the standard acute test condition (48-h CT), and then employed for all the following analyses.

### Cell Cycle Distribution and Cell Death

Concerning the cytofluorimetric analysis (Figure 1D), the graphics in Figure 1Da illustrate the DNA distribution in U251 control and 48-h CT cells exposed to 10 μM Pt(IV)Ac-POA. In the control condition, the cells were normally distributed among the different cell phases (G_1_, S, and G_2_). Differently, after 10 μM Pt(IV)Ac-POA 48-h CT, a slight decrease (14.73 ± 0.51%) of the S phase was observed. In appraising the cytograms, the presence of a sub-G_1_ peak was evidenced in the treated sample only, indicating high mortality of the cell population. Accordingly, the annexin V/PI staining confirmed that, compared to the control condition, after 10 μM Pt(IV)Ac-POA 48-h CT, an increase in the number of apoptotic cells was identified (Figure 1Db). In particular, the biparametric cytograms showed that the control (CTR) cell population was predominantly viable (97.51 ± 0.11%), with only 0.47 ± 0.09% and 1.22 ± 0.03% of early and late apoptotic cells, respectively. In contrast, in the treated sample, a strong reduction of living cells (50.13 ± 0.08%), paralleled by an augment of early (21.52 ± 0.12%) and late (25.74 ± 0.43%) apoptosis, was observed. A slight increase of necrotic cells (2.61 ± 0.04%) was also detected in the treated sample only.

### Ultrastructural Features by TEM

**FIGURE 2:**
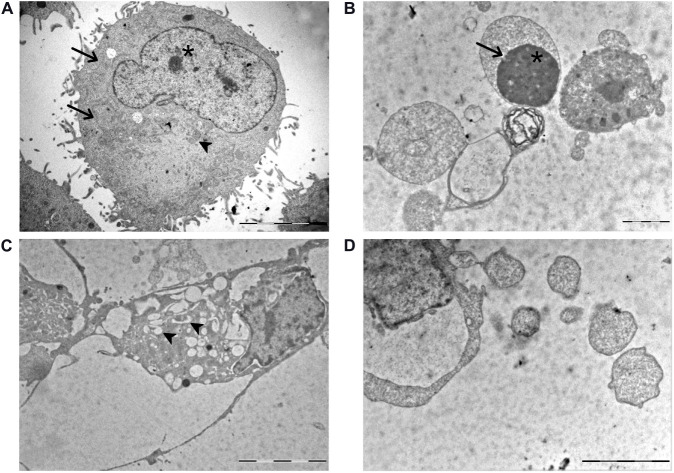
TEM ultrastructural analysis. **(A)** U251 control cells: *arrowhead* indicates the mitochondria with a physiological feature; *asterisk* identifies a well-visible nucleolus with a decondensed chromatin. A well-organized endoplasmic reticulum (ER) is also visible in the perinuclear zone (*thin arrows*). **(B–D)** U251 cells after 10 μM (*OC*-6-44)-acetatodiamminedichlorido(2-(2-propynyl)octanoato)platinum(IV) [Pt(IV)Ac-POA] 48-h continuous treatment (CT). **(B)** Chromatin condensation (*asterisk*) and absence of the nuclear envelope (*thin arrows*) in apoptotic cells. **(C)** Several vacuoles engulfed with cell debris (*arrowheads*) in autophagic cells. **(D)** Typical necroptotic features accompanied by a partially preserved cytoplasmic membrane. *Scale bar*, 5 μm.

Based on our previous *in vitro* findings (Gabano et al., 2017; Rangone et al., 2018; Ferrari et al., 2020) demonstrating the possible activation of different mechanisms after exposure to Pt(IV)Ac-POA, morphological changes were analyzed by electron microscopy. In the control condition, cells were characterized by the presence ofthe nucleus showing a decondensed chromatin, a well-organized endoplasmic reticulum (ER), located in the perinuclear area, and medium-sized mitochondria (Figure 2A), together with the presence of cytoplasmic protrusions around the cell body. Differently, after 10 μM Pt(IV)Ac-POA 48-h CT, different ultrastructural alterations were detected. Specifically, typical features representative of different cell death pathways were identified: (i) chromatin condensation and disappearance of the nuclear envelope, as a distinctive apoptosis hallmarks (Figure 2B); (ii) increased number of vacuoles containing cell debris, as a crucial element of autophagy (Figure 2C); and (iii) progressive chromatin condensation paralleled by a partially preserved cytoplasmic membrane, typically ascribable to necroptosis (Figure 2D).

### Apoptotic Cell Death Pathway Evaluation

Based on the distinctive features detected by TEM, the expressions and possible changes of the different proteins mainly involved in the diverse cell death pathways were assessed by immunofluorescence. Together with the standard acute test, the “REC” condition, previously mentioned in *Materials and Methods* (see section “Pharmacological Treatment and Exposure Conditions”), was included in these evaluations in order to reveal potential long-term defense strategies played by tumor cells several days after 10 μM Pt(IV)Ac-POA or 40 μM CDDP 48-h CT, during which the cells were maintained in drug-free normal EMEM. CDDP exposure was performed as a reference parameter, being the conventional treatment, already investigated in several studies (Grimaldi et al., 2016, 2019; Peng et al., 2018; Islam and Abdelilah Aboussekhra, 2019; Kuo et al., 2019).

**FIGURE 3:**
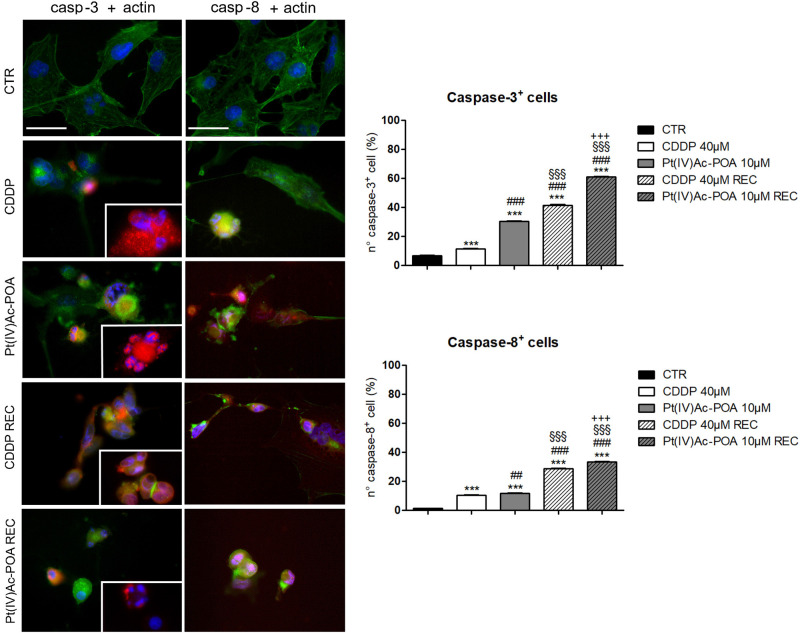
**Left panel:** Apoptotic pathway investigated by fluorescence microscopy in the controls, differently treated U251 cells, i.e., after 48-h continuous treatment (CT) with 40 μM *cis*-dichlorodiammineplatinum (CDDP) or 10 μM (*OC*-6-44)-acetatodiamminedichlorido(2-(2-propynyl)octanoato)platinum(IV) [Pt(IV)Ac-POA], and in the recovered (REC) conditions. Double immunolabeling for active caspase-3 and caspase-8 (*red fluorescence*) and actin (*green fluorescence*). Nuclear counterstaining with Hoechst 33258 (*blue fluorescence*). *Inserts*: high-magnification micrographs showing caspase-immunopositive nuclei and collapsed cytoskeleton. *Scale bar*, 40 μm. **Right panel:** Histograms showing the percentage value of caspase-3- and caspase-8-immunopositive cells, respectively. Statistical significance calculated as follows: ^∗^control *vs*. each experimental condition; ^#^CDDP *vs*. other treatments; ^§^Pt(IV)Ac-POA *vs*. REC conditions; **^+^**CDDP REC conditions *vs*. Pt(IV)AC-POA REC. ^∗∗∗^*p* < 0.001. ^###^*p* < 0.01; ^##^*p* < 0.001; ^§§§^*p* < 0.001; ^ + ⁣ + +^ *p* < 0.001.

The activation of the intrinsic and extrinsic apoptotic pathways was investigated focusing on cleaved caspase-3 and caspase-8, respectively (Figure 3). An increased immunopositivity was observed for both evaluated proteins, after both 10 μM Pt(IV)Ac-POA and 40 μM CDDP 48-h CT, compared to the control cells characterized by a lack of labeling. Notably, a progressive increase of immunofluorescence was detected in the REC samples when compared both to the controls as well as to their respective 48-h CT. Indeed, the greatest apoptogenic effect was observed in the REC samples, with the highest outcomes in those exposed to 10 μM Pt(IV)Ac-POA. The actin cytoskeleton immunolabeling revealed a well-defined structural organization in the control cells. In contrast, after both 10 μM Pt(IV)Ac-POA and 40 μM CDDP 48-h CT, some typical cellular degeneration features were detected, characterized by the cytoskeleton collapsing around the highly degraded nuclei. These alterations were also detectable in the Pt(IV)Ac-POA REC sample, while a partial restoration of normal cell morphology was observed in CDDP REC cells. The consequent quantitative measurements of caspase-3 and caspase-8 immunofluorescence confirmed an increased immunopositivity in cells after 10 μM Pt(IV)Ac-POA compared to both 40 μM CDDP 48-h CT and control conditions (Figure 3). A strong increase in caspase-3 and caspase-8 immunopositivity was also observed both in Pt(IV)Ac-POA REC samples as well as in CDDP REC cells, with the former showing the greatest increase in immunopositive cell density. The quantification values are summarized in Table 3 with their respective significance.

**TABLE 3:** Quantitative evaluation of cleaved caspases-3 and 8 immunopositivity.

***%***	***CTR***	***CDDP***	***Pt(IV)Ac-POA***	***CDDP REC***	***Pt(IV)Ac-POA REC***
*Casp-3*	6.66 ± 0.47	11.22 ± 0.27	30.48 ± 0.27	41.27 ± 0.57	61.02 ± 0.20
*Casp-8*	1.19 ± 0.09	10.34 ± 0.22	11.59 ± 0.27	28.57 ± 0.40	33.33 ± 0.34
**Caspase-3**					
**Bonferroni’s Multiple Comparison Test**	***ρ* value**				
CTR *vs.* 40 μM CDDP	***				
CTR *vs.* 10 μM Pt(IV)Ac-POA	***				
CTR *vs.* 40 μM CDDP *REC*	***				
CTR *vs.* 10 μM Pt(IV)Ac-POA *REC*	***				
40 μM CDDP *vs.* 10 μM Pt(IV)Ac-POA	***				
40 μM CDDP *vs.* 40 μM CDDP *REC*	***				
40 μM CDDP *vs.* 10 μM Pt(IV)Ac-POA *REC*	***				
10 μM Pt(IV)Ac-POA *vs.* 40 μM CDDP *REC*	***				
10 μM Pt(IV)Ac-POA *vs.* 10 μM Pt(IV)Ac-POA *REC*	***				
40 μM CDDP *REC vs.* 10 μM Pt(IV)Ac-POA *REC*	***				
**Caspase-8**					
**Bonferroni’s Multiple Comparison Test**	***ρ* value**				
CTR *vs.* 40 μM CDDP	***				
CTR *vs.* 10 μM Pt(IV)Ac-POA	***				
CTR *vs.* 40 μM CDDP *REC*	***				
CTR *vs.* 10 μM Pt(IV)Ac-POA *REC*	***				
40 μM CDDP *vs.* 10 μM Pt(IV)Ac-POA	*				
40 μM CDDP *vs.* 40 μM CDDP *REC*	***				
40 μM CDDP *vs.* 10 μM Pt(IV)Ac-POA *REC*	***				
10 μM Pt(IV)Ac-POA *vs.* 40 μM CDDP *REC*	***				
10 μM Pt(IV)Ac-POA *vs.* 10 μM Pt(IV)Ac-POA *REC*	***				
40 μM CDDP *REC vs.* 10 μM Pt(IV)Ac-POA *REC*	***				

*ρ < 0.05; **ρ < 0.01; ***ρ < 0.001.

**FIGURE 4:**
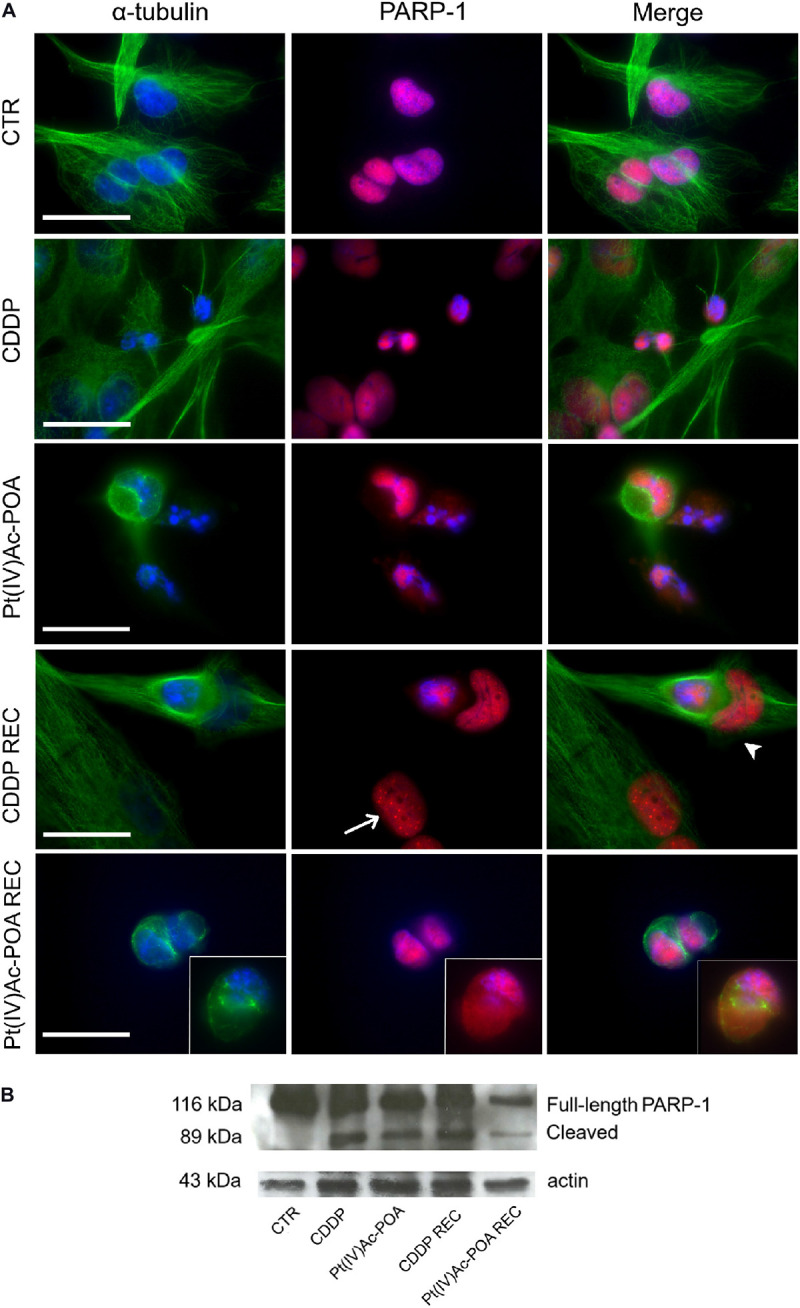
Apoptotic pathway investigated using fluorescence microscopy. **(A)** Double immunofluorescence detection of poly[ADP-ribose] polymerase 1 (PARP-1, *red fluorescence*) and α-tubulin (*green fluorescence*) in the controls, differently treated U251 cells, i.e., after 48-h continuous treatment (CT) with 40 μM *cis*-dichlorodiammineplatinum (CDDP) or 10 μM (*OC*-6-44)-acetatodiamminedichlorido(2-(2-propynyl)octanoato)platinum(IV) [Pt(IV)Ac-POA], and in the recovered (REC) conditions. DNA counterstaining with Hoechst 33258 (*blue fluorescence*). *Inserts*: high-magnification micrographs showing PARP-1 translocation from the nucleus to the cytoplasm. *Thin arrow* indicates PARP-1 “spot-like” labeling, while *arrowhead* designates phagocytic cell. Cytoskeletal alterations are also noticeable. *Scale bar*, 20 μm. **(B)** Western blotting data showing full-length PARP-1 (116 kDa) and cleaved PARP-1 (89 kDa) bands, respectively, compared to the loading control and actin (43 kDa).

Afterward, the activation of the cleaved caspase-3 substrate, i.e., poly[ADP-ribose] polymerase 1 (PARP-1), was investigated (Figure 4). In U251 control cells, PARP-1 was localized at the nuclear level, and the well-organized tubulin cytoskeleton ensured a normal cell morphology. After both 10 μM Pt(IV)Ac-POA and 40 μM CDDP 48-h CT, the cells underwent apoptosis, as indicated by the presence of several degraded nuclei. Interestingly, different cell death phases were observed: (i) late apoptosis, characterized by the lack of PARP-1 immunofluorescence in the nuclei, and (ii) early apoptosis, in which PARP-1, or rather the p89 fragment, moved from the nucleus to the cytoplasm. This latter phenomenon was especially evident after 10 μM Pt(IV)Ac-POA. On the other hand, after 40 μM CDDP 48-h CT, cells with a healthy phenotype still showed a nuclear PARP-1 signal. In the CDDP REC condition, the fluorescent “spot-like” PARP-1 signal was observed. In this condition, apoptosis was also observed, paralleled by some features resembling an ongoing phagocytosis process. In the Pt(IV)Ac-POA REC condition, apoptosis occurrence was observed in several cells, while some cells, presenting PARP-1 nuclear expression, displayed a strongly altered tubulin cytoskeleton, probably indicating possible cellular damage (Figure 4A). Lastly, Western blot analyses showed PARP-1 activation after both chemotherapeutic treatments, considering all exposure conditions, compared to the controls. It should be noted that in the Pt(IV)Ac-POA REC condition, an evident reduction in PARP-1 expression was detected, nonetheless associated with its activation. This data could suggest a strong, long-lasting Pt(IV)Ac-POA-induced effect in reducing PARP-1 expression, this protein being pivotally involved in DNA repair (Figure 4B).

**FIGURE 5:**
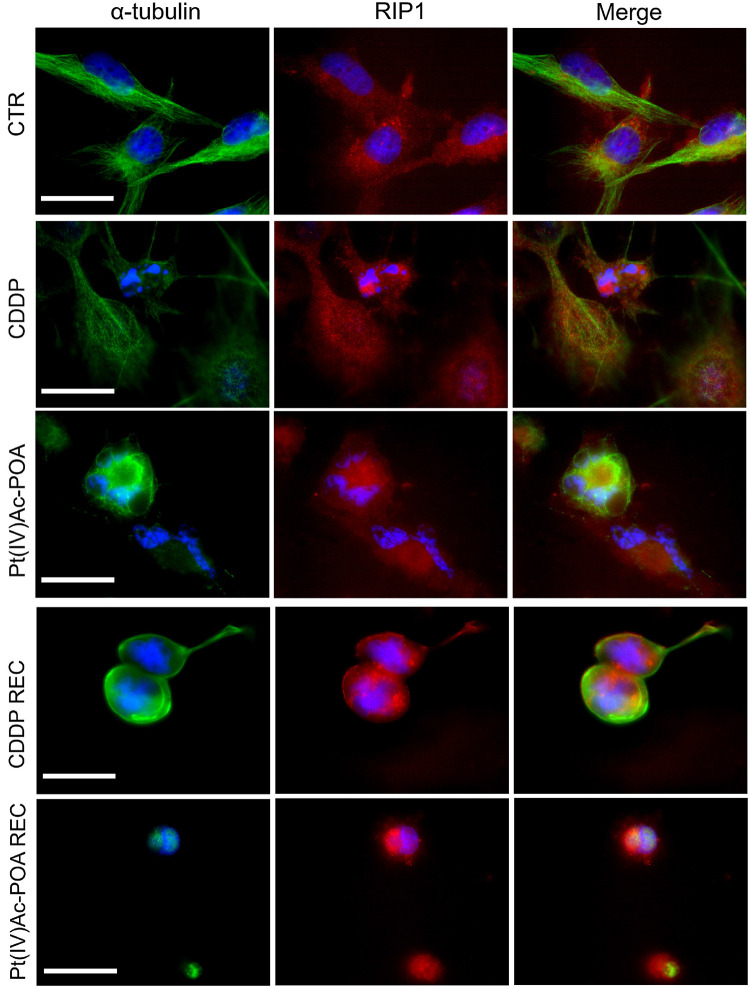
Extrinsic apoptotic pathway evaluation by double immunocytochemical detection of receptor-interacting protein kinase 1 (RIP1, *red fluorescence*) and α-tubulin (*green fluorescence*) in the controls, differently treated U251 cells, i.e., after 48-h continuous treatment (CT) with 40 μM *cis*-dichlorodiammineplatinum (CDDP) or 10 μM (*OC*-6-44)- acetatodiamminedichlorido(2-(2-propynyl)octanoato)platinum(IV) [Pt(IV)Ac-POA], and in the recovered (REC) conditions. DNA counterstaining with Hoechst 33258 (*blue fluorescence*). *Scale bar*, 20 μm.

Concerning caspase-8, a critical mediator of the extrinsic apoptotic pathway, the RIP1 (receptor-interacting protein kinase 1) protein expression and changes were investigated by immunofluorescence to confirm the activation of this apoptotic pathway and the possible preliminary activation of necroptosis (Figure 5). In control cells, RIP1 fluorescence signal was homogeneously detectable in the entire cytoplasm, but being completely lacking at the nuclear level. After 40 μM CDDP 48-h CT, a rearrangement of the fluorescence signal was observed, mainly localized around the fragmented nuclei in the majority of cells; a RIP1 cytoplasmic expression was perceived only in some clusters of cells displaying characteristics similar to those observed in the control condition. Notably, after 10 μM Pt(IV)Ac-POA, a significant increase in RIP1 signal was identified around the degraded nuclei. Furthermore, the tubulin cytoskeleton appeared strongly damaged compared to both the control and CDDP conditions. In the REC condition, after both treatments, non-viable cells were observed, with a marked increase in RIP1 fluorescence signal, mostly concentrated around the visibly damaged nuclei. In particular, in the Pt(IV)Ac-POA REC condition, cells displayed evident pyknotic features.

**FIGURE 6:**
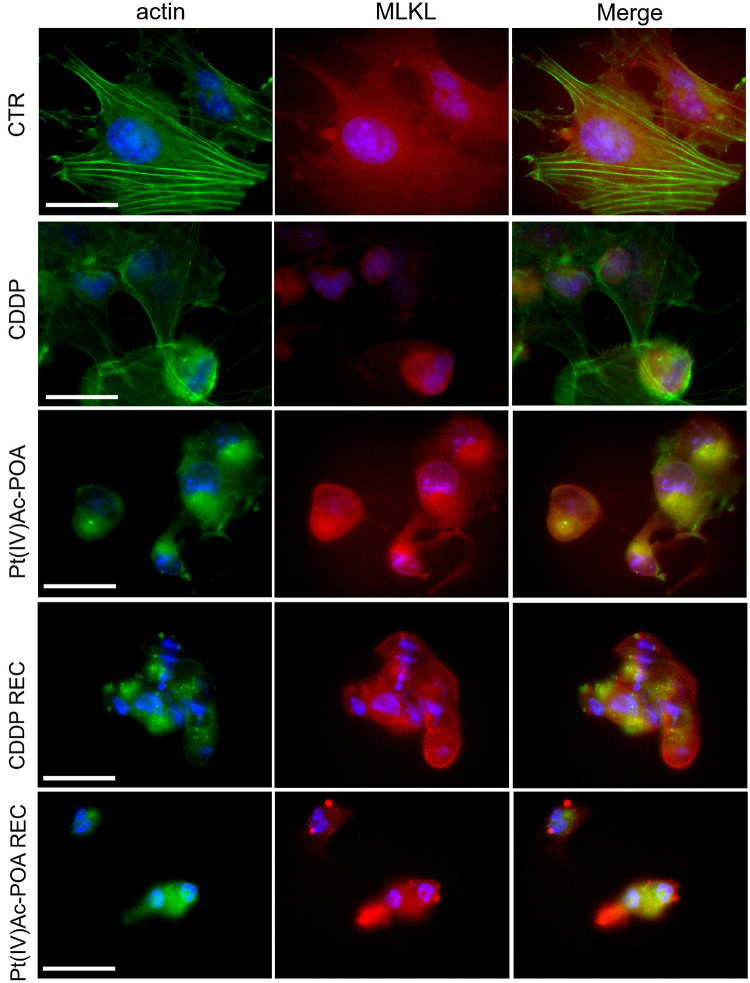
Investigation of necroptotic pathway using fluorescence microscopy in the controls, differently treated U251 cells, i.e., after 48-h continuous treatment (CT) with 40 μM *cis*-dichlorodiammineplatinum (CDDP) or 10 μM (*OC*-6-44)-acetatodiamminedichlorido(2-(2-propynyl)octanoato) platinum(IV) [Pt(IV)Ac-POA], and in the recovered (REC) conditions. Double immunolabeling detection of mixed lineage kinase domain-like (MLKL, *red fluorescence*) and actin (*green fluorescence*). DNA counterstaining with Hoechst 33258 (*blue fluorescence*). *Scale bar*, 20 μm.

Based on the knowledge that (i) in necrosome formation, RIP1, or rather RIP3, interacts with mixed lineage kinase domain-like (MLKL) that is essential to induce necroptosis and (ii) MLKL conformational change promotes its translocation to the plasma membrane, causing its permeabilization, but also to the nucleus in the early necroptosis stage (Weber et al., 2018), the possible MLKL translocation was presently investigated (Figure 6). In control cells, cytoplasmic labeling was detected, while after both acute drug treatments the MLKL fluorescence signal was rearranged in cells characterized by evident fragmented nuclei. After 40 μM CDDP 48-h CT, the MLKL signal was localized around the damaged nuclei. A similar effect was observed after 10 μM Pt(IV)Ac-POA 48-h CT; besides, in this condition, a strong alteration of the actin cytoskeleton was detectable. After 10 μM Pt(IV)Ac-POA 48-h CT and in the REC condition after either CDDP or Pt(IV)Ac-POA, a significant increase of MLKL expression was detected, particularly evident in cells showing overt altered features. The accumulation of MLKL fluorescence in the degraded cell nuclei of the Pt(IV)Ac-POA REC sample has to be noted.

**FIGURE 7:**
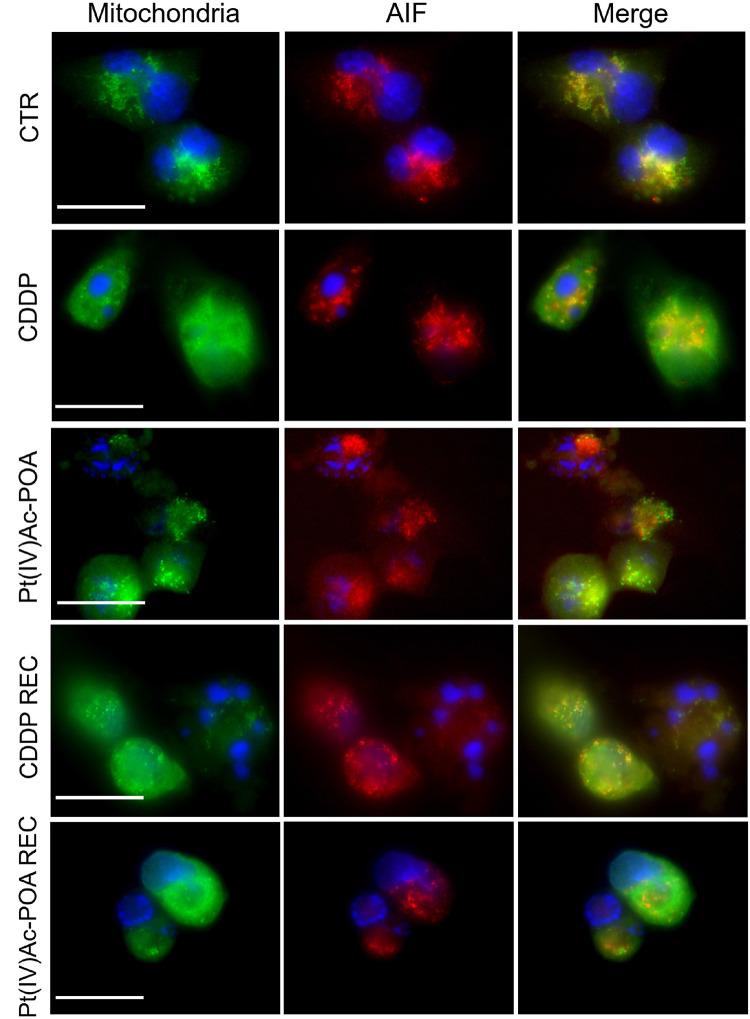
Caspase-independent cell death pathway activation investigated by fluorescence microscopy. Double immunostaining for mitochondria (*green fluorescence*) and apoptosis-inducing factor (AIF, *red fluorescence*) in the controls, differently treated U251 cells, i.e., after 48-h continuous treatment (CT) with 40 μM *cis*-dichlorodiammineplatinum (CDDP) or 10 μM (*OC*-6-44)-acetatodiamminedichlorido(2-(2-propynyl)octanoato)platinum(IV) [Pt(IV)Ac-POA], and in the recovered (REC) conditions. DNA counterstaining with Hoechst 33258 (*blue fluorescence*). *Scale bar*, 20 μm.

Subsequently, the activation of the caspase-independent cell death pathway was investigated by immunofluorescence, focusing on the apoptosis-inducing factor (AIF) protein and the mitochondria (Figure 7). In control cells, AIF expression was observed at the mitochondria level, as sustained by the colocalization of the two fluorescence signals. Following 48-h CT with either 40 μM CDDP or 10 μM Pt(IV)Ac-POA, AIF progressively lost its physiological localization, moving into the cytoplasm and then into the nucleus.

After 40 μM CDDP 48-h CT or 10 μM Pt(IV)Ac-POA 48-h CT, the translocation of AIF to the nucleus was particularly evident. This effect was particularly manifest in cells treated with Pt(IV)Ac-POA, in which the loss of colocalization of the two fluorescence signals was evident. Diversely, in the CDDP REC condition, the AIF translocation process was less discernible, suggesting a possible return to the normal mitochondrial localization, which could, therefore, be associated with a cellular recovery mechanism. In contrast, in the Pt(IV)Ac-POA REC sample, AIF remained in the perinuclear area without any sign of restoration of the physiological mitochondrial localization.

### Autophagic Pathway Activation

**FIGURE 8:**
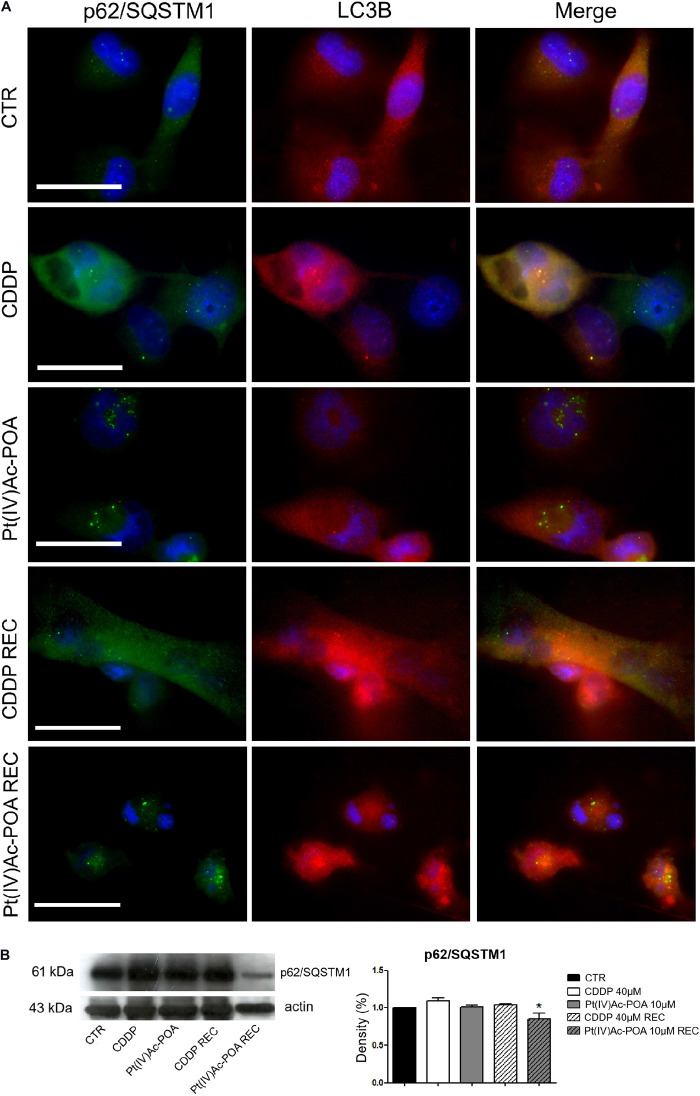
Immunofluorescence study of autophagy activation. **(A)** Double immunolabeling for p62/SQSTM1 (*green fluorescence*) and LC3B (*red fluorescence*). DNA counterstaining with Hoechst 33258 (*blue fluorescence*). *Scale bar*, 20 μm. **(B)** Western blotting data: p62/SQSTM1. Histograms representing density band quantification of p62/SQSTM1 in the controls, differently treated U251 cells, i.e., after 48-h continuous treatment (CT) with 40 μM *cis*-dichlorodiammineplatinum (CDDP) or 10 μM (*OC*-6-44)-acetatodiamminedichlorido(2-(2-propynyl)octanoato)platinum(IV) [Pt(IV)Ac-POA], and in the recovered (REC) conditions. Statistical significance: *ρ < 0.05, 40 μM CDDP 48-h CT *vs*. the Pt(IV)AC-POA REC condition.

To deepen the above-reported results revealing the presence of typical autophagic features, p62/SQSTM1 and LC3B, being two specific markers of autophagy, were then evaluated. In control cells, the two fluorescence signals did not colocalize; in particular, p62/SQSTM1 was detected both in the cytoplasm and the nucleus, while LC3B was predominantly identified in the cytoplasm. Differently to the condition observed after 10 μM Pt(IV)Ac-POA 48-h CT, which was similar to that observed in the controls, after 40 μM CDDP 48-h CT, the colocalization of the two protein signals was detectable, suggesting an activation of the autophagic process. Notably, in the REC condition after CDDP only, the cells displayed a strongly modified morphology, showing an increased size and a significant enhancement of the basal expressions of both p62/SQSTM1 and LC3B, whose fluorescence signals did not colocalize (Figure 8A). Differently, the Pt(IV)Ac-POA REC samples exhibited a degraded morphology associated with the colocalization of the two fluorescent markers. Further, Western blotting analyses (Table 4) demonstrated that, despite a significant reduction in the expression levels of p62/SQSTM1 when comparing the Pt(IV)Ac-POA REC condition to 40 μM CDDP 48-h CT, any statistically significant difference was obtained comparing the control to all other treatments/exposure conditions (Figure 8B).

**TABLE 4:** Protein (p62/SQSTM1) band density quantification after Western blotting experiments.

***%***	***CTR***	***CDDP***	***Pt(IV)Ac-POA***	***CDDP REC***	***Pt(IV)Ac-POA REC***
***p62/SQSTM1***	1.00 ± 0.00	1.09 ± 0.04	1.01 ± 0.02	1.04 ± 0.02	0.85 ± 0.08

### Pt(IV)Ac-POA Effects on Cytoplasmic Organelles

Previous investigations demonstrated that platinum compounds may affect intracellular organelles. Experimental studies using different neuronal cell lineages highlighted a significant effect of cisplatin and platinum(II)-based compound on the Golgi apparatus and mitochondria (Santin et al., 2012, 2013; Grimaldi et al., 2016; Ferrari et al., 2020). Therefore, in the present study, we explored whether the above-mentioned cytoplasmic organelles may be a target of the novel Pt(IV)Ac-POA.

In control cells, Golgi apparatus immunofluorescence showed a normal appearance, with homogeneous distribution in the perinuclear zone and, partially, throughout the cell body. Additionally, the actin-positive cytoskeleton maintained the normal shape and internal organization.

**FIGURE 9:**
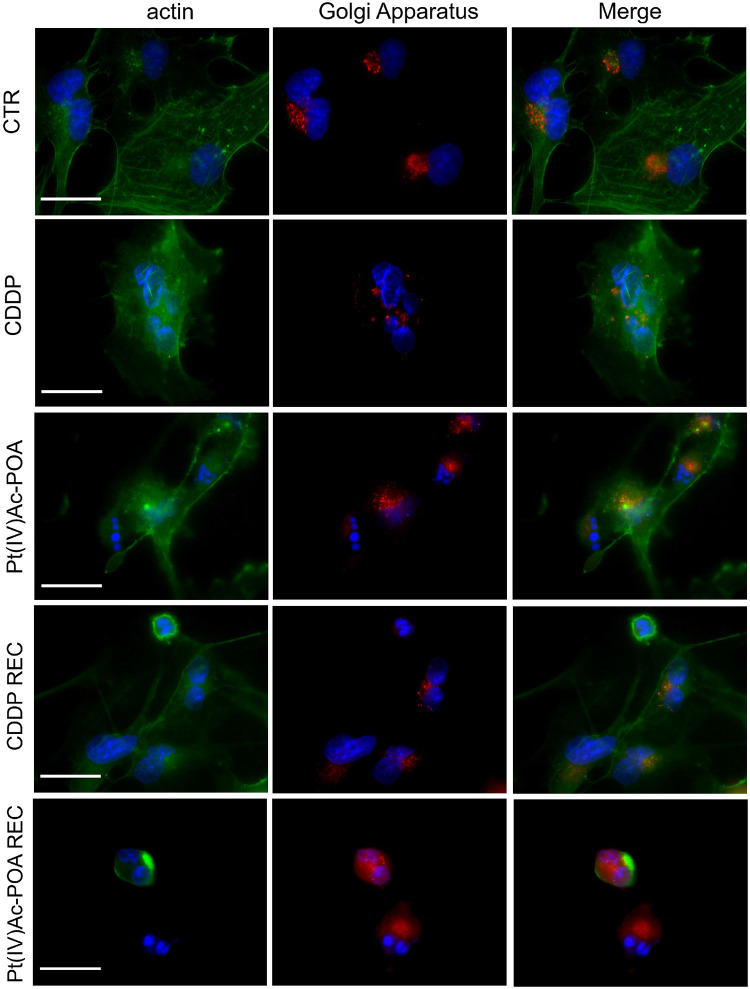
Intracellular organelle investigation by immunofluorescence. Double immunocytochemical detection of the Golgi apparatus (*red fluorescence*) and actin (*green fluorescence*) in the controls, differently treated U251 cells, i.e., after 48-h continuous treatment (CT) with 40 μM *cis*-dichlorodiammineplatinum (CDDP) or 10 μM (*OC*-6-44)- acetatodiamminedichlorido(2-(2-propynyl)octanoato)platinum(IV) [Pt(IV)Ac-POA], and in the recovered (REC) conditions. DNA counterstaining with Hoechst 33258 (*blue fluorescence*). *Scale bars*, 20 μm.

In contrast, after 40 μM CDDP 48-h CT or 10 μM Pt(IV)Ac-POA 48-h CT, a degeneration of Golgi cistern arrangement and cytoskeletal structure was detected. In detail, a degradation of the Golgi apparatus occurred, resulting in the presence of diffuse and homogeneous immunofluorescence localized around the fragmented nuclei. This effect was significantly more marked in the Pt(IV)Ac-POA REC samples, where the cytoskeleton and Golgi apparatus were even no longer distinguishable. Differently, in the CDDP REC condition, despite the presence of some shrunken cells, restoration of physiological features was perceived, resembling the characteristics observed in the control condition. Specifically, a well-defined actin cytoskeletal immunofluorescence as well as the Golgi apparatus immunolabeling were clearly observable (Figure 9).

**FIGURE 10:**
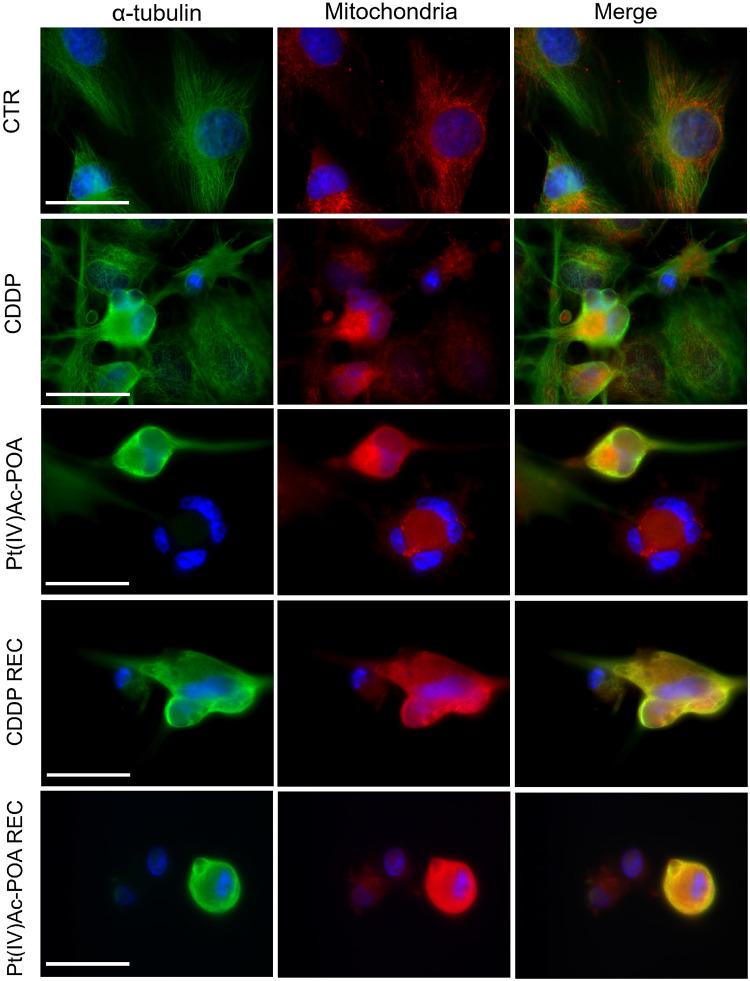
Intracellular organelle investigation by immunofluorescence. Double immunolabeling for the mitochondria (*red fluorescence*) and α-tubulin (*green fluorescence*) in the controls, differently treated U251 cells, i.e., after 48-h continuous treatment (CT) with 40 μM *cis*-dichlorodiammineplatinum (CDDP) or 10 μM (*OC*-6-44)-acetatodiamminedichlorido(2-(2-propynyl) octanoato)platinum(IV) [Pt(IV)Ac-POA], and in the recovered (REC) conditions. DNA counterstaining with Hoechst 33258 (*blue fluorescence*). *Scale bars*, 20 μm.

A similar trend was observed when evaluating the double immunofluorescence for the mitochondria and tubulin cytoskeleton (Figure 10). In the control conditions, the mitochondria were evenly distributed throughout the entire cell cytoplasm, arranged according to the microtubule distribution pattern of the well-organized cytoskeleton. After 10 μM Pt(IV)Ac-POA 48-h CT, a striking damaging effect was detected, with the mitochondria showing a small and rounded organization, being often clustered within dying cells. This effect retained a long-lasting duration, being even measurable in the respective REC condition. After 40 μM CDDP 48-h CT, several cells displayed characteristics similar to those observed in controls; nonetheless, a worsened situation was perceived in the respective REC condition. Notably, after all different exposure conditions, CDDP treatment was demonstrated to cause less intense effects than those measured after exposure to Pt(IV)Ac-POA.

### Irradiation With Carbon Ions

Initial experimental studies have then been conducted on U251 cells using carbon ion irradiation. The effects were assessed using different experimental conditions, with the goal to investigate the action of hadrontherapy alone and combined with the two chemotherapeutics under investigation, i.e., CDDP and Pt(IV)Ac-POA. The same drug concentrations previously employed for the above reported *in vitro* experiments were employed in order to compare the effects caused by the combined protocol (drug exposure + hadrontherapy) with those obtained after chemotherapeutic treatments alone. Specifically, clonogenic cell survival as well as apoptotic and autophagic markers, already determined after chemotherapeutic therapy alone, were assessed after 48-h CT to either CDDP or Pt(IV)Ac-POA followed by carbon ion irradiation at doses of 2 and 4 Gy. Preliminary data concerning the REC conditions were also obtained.

#### Clonogenic Cell Survival Assay: Standard Acute Treatment With 10 μM Pt(IV)Ac-POA Plus Increasing Carbon Ion Irradiation Doses

Compared to the data obtained after exposure to the prodrug alone, the clonogenicity of U251 was further impaired when exposed to the combined treatment, i.e., 10 μM Pt(IV)Ac-POA + carbon ion irradiation at increasing doses (i.e. 1, 2, and 4 Gy). Specifically, a synergistic effect was measured when 10 μM Pt(IV)Ac-POA was combined with two of the tested radiation doses, i.e., 2 and 4 Gy, reducing to the colony formation of about 85%, with surviving fractions of 16 and 13% after 2 and 4 Gy, respectively (Figures 1Cb,d). Notably, these data demonstrated that the synergistic effect played by the combined treatment with 10 μM Pt(IV)Ac-POA + carbon ion radiation was similar using either 2 or 4 Gy. Diversely, the combined exposure to 10 μM Pt(IV)Ac-POA + 1 Gy induced a clonogenicity impairment similar to that observed after treatment with the prodrug alone (26% *vs*. 30%) (Figures 1Cb,d). These findings supported that the clonogenicity of glioblastoma cells was impaired synergistically when Pt(IV)Ac-POA was combined with hadrontherapy.

#### Acute Exposure Condition—Apoptotic Pathway Evaluation: Caspase-3 and PARP-1 Immunofluorescence Staining

**FIGURE 11:**
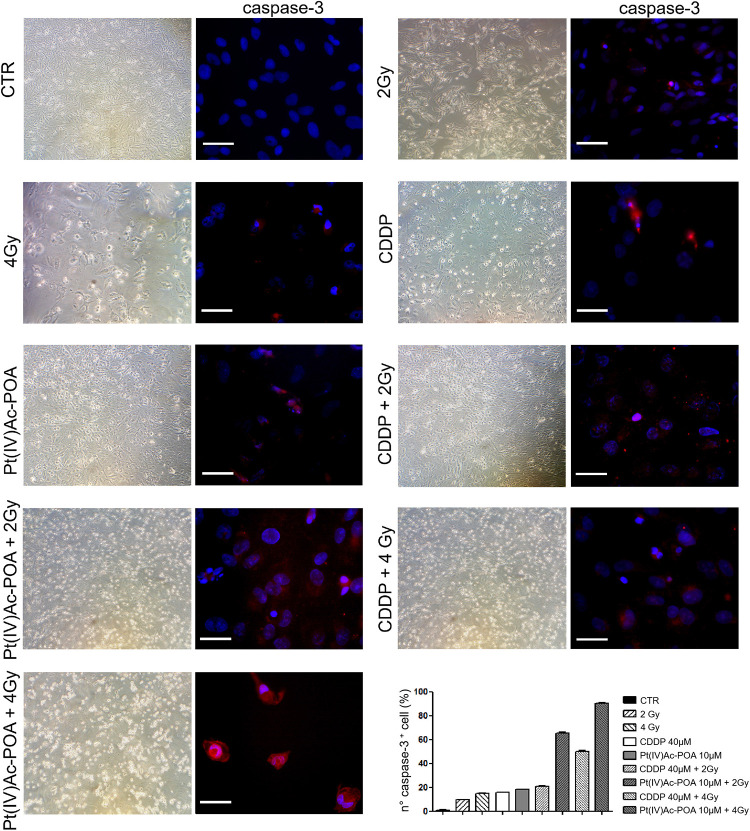
Different experimental conditions: chemotherapeutics exposure alone and followed by carbon ion irradiation. The **left column** shows phase-contrast micrographs of U251 cells cultured in flask sterile on slide in different experimental conditions before being processed for immunofluorescence reactions. The **right column** displays caspase-3 immunolabeling (*red fluorescence*) in the control (CTR), differently treated U251 cells, i.e., after 48-h continuous treatment (CT) with 40 μM *cis*-dichlorodiammineplatinum (CDDP) or 10 μM (*OC*-6-44)-acetatodiamminedichlorido(2-(2-propynyl)octanoato)platinum(IV) [Pt(IV)Ac-POA] alone or followed by carbon ion radiation at 2 or 4 Gy dose. DNA counterstaining with Hoechst 33258 (*blue fluorescence*). *Scale bar*, 40 μm. Histograms (**lower right part**) representing the percentage values of caspase-3-immunopositive cells.

As illustrated in Figure 11, caspase-3 immunofluorescence increased in the samples exposed to carbon ion radiation (10.71 ± 0.02% and 15.00 ± 0.31% for 2 and 4 Gy, respectively) compared to the controls (0.94 ± 0.15%). Compared to the former samples, immunopositivity slightly increased after 40 μM CDDP 48-h CT (15.71 ± 0.30%) or 10 μM Pt(IV)Ac-POA 48-h CT (18.12 ± 0.39%). Notably, the number of caspase-3-positive cells significantly increased after the combined treatment, i.e., chemotherapeutics exposure followed by carbon ion radiation. Specifically, the samples firstly treated with 40 μM CDDP or 10 μM Pt(IV)Ac-POA and then exposed to 4 Gy showed a strong increase of caspase-3 immunofluorescence (50.00 ± 0.99% and 90.24 ± 0.47%, respectively) compared to the cells exposed to drug treatments followed by the 2 Gy dose (20.83 ± 0.43% and 65.16 ± 0.94%, respectively). In detail, the greatest effect was measured in the samples exposed to Pt(IV)Ac-POA followed by carbon ion radiation at a dose of 4 Gy. Table 5 summarizes the significant values of caspase-3-immunopositive cell quantification.

**TABLE 5:** Caspase-3 immunofluorescence: summary of statistical significances.

**Bonferroni’s Multiple Comparison Test**	***ρ* value**
CTR *vs.* 2 Gy	***
CTR *vs.* 4 Gy	***
CTR *vs.* 40 μM CDDP	***
CTR *vs.* 10 μM Pt(IV)Ac-POA	***
CTR *vs.* 40 μM CDDP + 2 Gy	***
CTR *vs.* 10 μM Pt(IV)Ac-POA + 2 Gy	***
CTR *vs.* 40 μM CDDP + 4 Gy	***
CTR *vs.* 10 μM Pt(IV)Ac-POA + 4 Gy	***
2 Gy *vs.* 4 Gy	***
2 Gy *vs.* 40 μM CDDP	***
2 Gy *vs.* 10 μM Pt(IV)Ac-POA	***
2 Gy *vs.* 40 μM CDDP + 2 Gy	***
2 Gy *vs.* 10 μM Pt(IV)Ac-POA + 2 Gy	***
2 Gy *vs.* 40 μM CDDP + 4 Gy	***
2 Gy *vs.* 10 μM Pt(IV)Ac-POA + 4 Gy	***
4 Gy *vs.* 40 μM CDDP	*ns*
4 Gy *vs.* 10 μM Pt(IV)Ac-POA	**
4 Gy *vs.* 40 μM CDDP + 2 Gy	***
4 Gy *vs.* 10 μM Pt(IV)Ac-POA + 2 Gy	***
4 Gy *vs.* 40 μM CDDP + 4 Gy	***
4 Gy *vs.* 10 μM Pt(IV)Ac-POA + 4 Gy	***
40 μM CDDP *vs.* 10 μM Pt(IV)Ac-POA	*ns*
40 μM CDDP *vs.* 40 μM CDDP + 2 Gy	***
40 μM CDDP *vs.* 10 μM Pt(IV)Ac-POA + 2 Gy	***
40 μM CDDP *vs.* 40 μM CDDP + 4 Gy	***
40 μM CDDP *vs.* 10 μM Pt(IV)Ac-POA + 4 Gy	***
10 μM Pt(IV)Ac-POA *vs.* 40 μM CDDP + 2 Gy	*
10 μM Pt(IV)Ac-POA *vs.* 10 μM Pt(IV)Ac-POA + 2 Gy	***
10 μM Pt(IV)Ac-POA *vs.* 40 μM CDDP + 4Gy	***
10 μM Pt(IV)Ac-POA *vs.* 10 μM Pt(IV)Ac-POA + 4 Gy	***
40 μM CDDP + 2 Gy *vs.* 10 μM Pt(IV)Ac-POA + 2 Gy	***
40 μM CDDP + 2 Gy *vs.* 40 μM CDDP + 4 Gy	***
40 μM CDDP + 2 Gy *vs.* 10 μM Pt(IV)Ac-POA + 4 Gy	***
10 μM Pt(IV)Ac-POA + 2 Gy *vs.* 40 μM CDDP + 4 Gy	***
10 μM Pt(IV)Ac-POA + 2 Gy *vs.* 10 μM Pt(IV)Ac-POA + 4 Gy	***
40 μM CDDP + 4 Gy *vs.* 10 μM Pt(IV)Ac-POA + 4 Gy	***

Different experimental conditions (CTR, control, 40 μM CDDP and 10 μM Pt(IV)Ac-POA 48 h-CT alone or combined with 2 or 4 Gy dose irradiation); *ρ* values: *ρ < 0.05; **ρ < 0.01; ***ρ < 0.001, *ns*, not significant.

**FIGURE 12:**
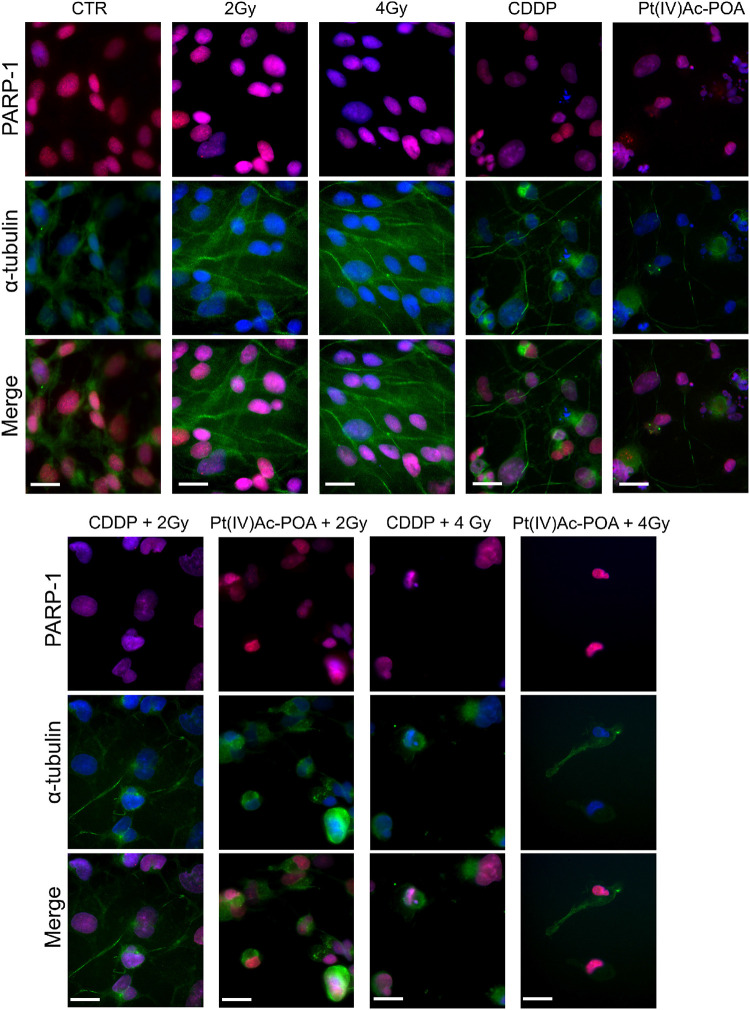
Apoptotic pathway investigated using immunocytochemistry after treatment with chemotherapeutics alone and combined with carbon ion irradiation at different doses. Double immunofluorescence detection of poly[ADP-ribose] polymerase 1 (PARP-1, *red fluorescence*) and α-tubulin (*green fluorescence*) in the controls, differently treated U251 cells, i.e., after 48-h continuous treatment (CT) with 40 μM *cis*-dichlorodiammineplatinum (CDDP) or 10 μM (*OC*-6-44)-acetatodiamminedichlorido(2-(2-propynyl)octanoato)platinum(IV) [Pt(IV)Ac-POA], and in combined exposure conditions [40 μM CDDP or 10 μM Pt(IV)Ac-POA 48-h CT + 2 or 4 Gy carbon ion radiation]. DNA counterstaining with Hoechst 33258 (*blue fluorescence*). *Scale bar*, 20 μm.

**FIGURE 13:**
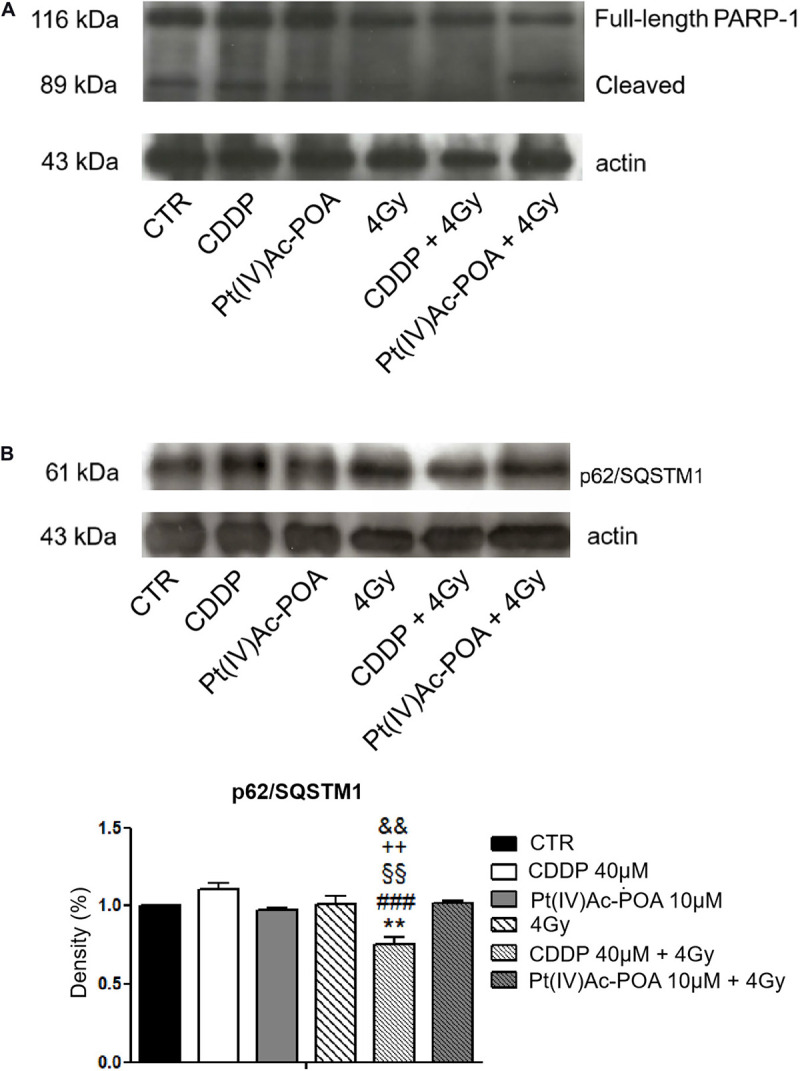
**(A)** Western blotting analysis of full-length poly[ADP-ribose] polymerase 1 (PARP-1, 116 kDa) and cleaved PARP-1 (89 kDa). Representative Western blotting bands showing the expression levels of full-length PARP-1 (116 kDa) and cleaved PARP-1 in the controls, differently treated U251 cells, i.e., after 48-h continuous treatment (CT) with 40 μM *cis*-dichlorodiammineplatinum (CDDP) or 10 μM (*OC*-6-44)-acetatodiamminedichlorido(2-(2-propynyl)octanoato)platinum(IV) [Pt(IV)Ac-POA], after carbon ion irradiation (4 Gy dose) alone, and in combined exposure conditions [40 μM CDDP or 10 μM Pt(IV)Ac-POA 48-h CT + 4 Gy carbon ion irradiation]. The result was quantified from the average of three different samples. The density bands of full-length (116 kDa) and cleaved (89 kDa) PARP-1 were compared to the loading control and actin (43 kDa). **(B)** Western blotting study of p62/SQSTM1 as a typical marker of autophagy activation. Histograms representing density band quantification of p62/SQSTM1 (61 kDa) in the controls, differently treated U251 cells, i.e., after 48-h CT with 40 μM CDDP or 10 μM Pt(IV)Ac-POA, after carbon ion radiation (4 Gy dose) alone, and in combined exposure conditions [40 μM CDDP or 10 μM Pt(IV)Ac-POA 48-h CT + 4 Gy carbon ion radiation]. The means, obtained from three independent experiments, have been quantified and compared to the loading control and actin (43 kDa). Statistical significance calculated as follows: *control *vs*. CDDP + 4 Gy carbon ion radiation; ^#^CDDP *vs*. CDDP + 4 Gy carbon ion radiation; **^§^** Pt(IV)Ac-POA *vs*. CDDP + 4 Gy carbon ion radiation; **^+^**4 Gy carbon ion radiation alone *vs*. CDDP + 4 Gy carbon ion radiation; **^&^**CDDP + 4 Gy carbon ion radiation *vs*. Pt(IV)Ac-POA + 4 Gy carbon ion radiation. **p < 0.01; ^###^*p* < 0.001; ^§§^*p* < 0.01; ^ + +^ *p* < 0.01; ^&&^*p* < 0.01.

The results pertaining to PARP-1 expression are shown in Figure 12. In control cells, PARP-1 was localized at the nuclear level, and the well-organized tubulin cytoskeleton supported the maintenance of the normal cell morphology. A similar immunopositivity trend was detected in the samples exposed to carbon ion radiation alone (applying both 2 and 4 Gy doses), in which PARP-1 immunofluorescence was observed in the nuclei accompanied by the presence of an organized tubulin cytoskeleton. After 40 μM CDDP 48-h CT or 10 μM Pt(IV)Ac-POA 48-h CT, various cells displayed degraded nuclei and a loss of their usual elongated shape due to cytoskeletal alterations. Interestingly, the cells treated with Pt(IV)Ac-POA alone or combined with carbon ion exposure showed different cell death phases, i.e., early and late apoptosis. Differently, the cells exposed to CDDP alone conserved a nuclear PARP-1 immunofluorescence signal. The most striking morphological alterations were identified in the samples exposed to combined therapy, i.e., early exposure to the compounds followed by ion radiation. After 40 μM CDDP 48-h CT associated with 2 Gy radiation, any marked increase in the cells with degraded nuclei was measured; nevertheless, the cytoskeleton showed structural abnormalities. Notably, when the CDDP treatment was associated with 4 Gy radiation dose, the effect was amplified, distressing not only the cytoskeleton but also the nucleus. The greatest cytotoxic effect was, however, observed after 10 μM Pt(IV)Ac-POA 48-h CT followed by 2 or 4 Gy radiation exposure. Important morphological alterations were detected already at 2 Gy, and the effect lasted and increased even at the 4-Gy dose. Based on the significant data obtained after radiation exposure at 4 Gy, this dose was selected to conduct Western blot determinations (Figure 13A). Accordingly to the above reported data concerning the first experimental step, Western blotting analyses demonstrated, yet again, no significant difference in the PARP-1 expression levels (including the cleaved fragment p89) in the cells either exposed to CDDP or Pt(IV)Ac-POA after 48-h CT. Notably, U251 irradiated with the 4-Gy dose alone, as well as the samples exposed to 40 μM CDDP 48-h CT followed by 4 Gy carbon ion radiation, showed similar full-length PARP-1 expression patterns; differently, cleaved PARP-1 was detected in 4-Gy-irradiated cells only. In these latter conditions, the expression of the cleaved PARP-1 fragment was strongly decreased compared to the cells exposed either to 40 μM CDDP 48-h CT or to 10 μM Pt(IV)Ac-POA 48-h CT. In the cells exposed to 10 μM Pt(IV)Ac-POA followed by 4 Gy carbon ion radiation, a reduced expression of full-length PARP-1 was observed paralleled by the presence of cleaved PARP-1.

#### Acute Exposure Condition—Autophagic Pathway Activation: p62/SQSTM1 Expression

Based on the significant data obtained after radiation exposure at 4 Gy, this dose was selected to conduct subsequent Western blotting analyses. The obtained data, shown in Figure 13B, demonstrated an increased, though not statistically significant, expression of p62/SQSTM1 protein in the CDDP-treated sample compared to the controls, suggesting a homeostatic imbalance in the activation of the autophagic pathway. On the contrary, when 40 μM CDDP 48-h CT was followed by 4 Gy radiation exposure, a significant decrease of the p62/SQSTM1 protein expression was measured, indicating the protein involvement in autophagy activation. In the sample exposed to 4 Gy radiation alone, the cells did not display any appreciable difference compared to the controls. After 10 μM Pt(IV)Ac-POA 48-h CT and after the combined exposure to 10 μM Pt(IV)Ac-POA 48-h CT + 4 Gy, the cells showed a p62/SQSMT1 level similar to that observed in the controls. Table 6 summarizes the Western blotting quantitative data.

**TABLE 6:** Protein (p62/SQSTM1) band density quantification after Western blotting experiments.

**%**	**CTR**	**CDDP**	**Pt(IV)Ac-POA**	**4Gy**	**CDDP + 4Gy**	**Pt(IV)Ac-POA + 4Gy**
**p62/SQSTM1**	1.00 ± 0.00	1.11 ± 0.03	0.93 ± 0.02	1.01 ± 0.06	0.75 ± 0.04	1.02 ± 0.02

#### REC Conditions: Apoptotic Pathway Evaluation: Caspase-3 and PARP-1 Assessment

**FIGURE 14:**
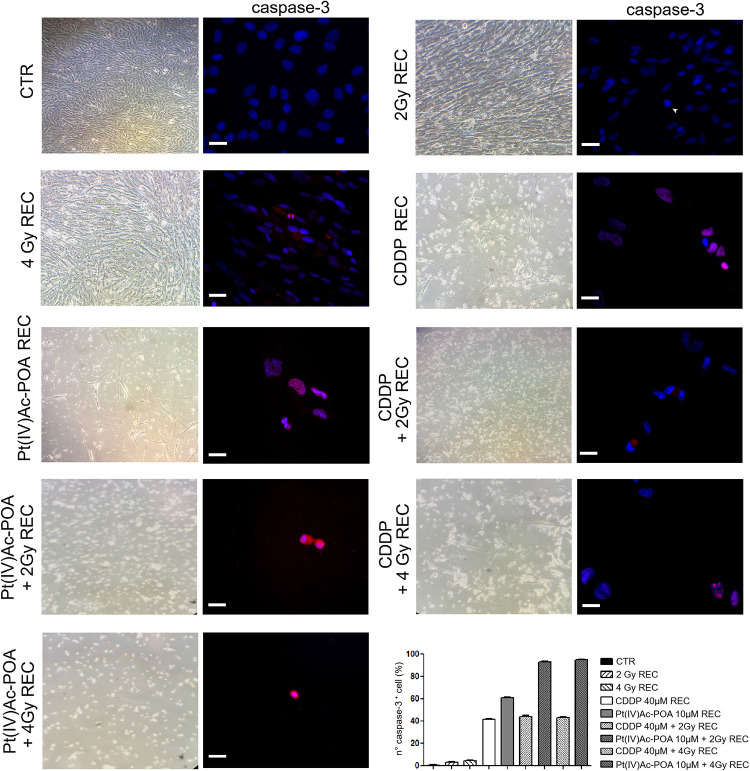
Recovered (REC) conditions after combined experimental protocol (chemotherapeutics exposure + carbon ion radiation). The **left column** shows phase-contrast micrographs of U251 cells cultured in flask sterile on slide in different experimental conditions before being processed for immunofluorescence reactions. The **right column** displays caspase-3 immunolabeling (*red fluorescence*) in the controls (CTR) and all different REC conditions, i.e., after 48-h continuous treatment (CT) with 40 μM *cis*-dichlorodiammineplatinum (CDDP) or 10 μM (*OC*-6-44)-acetatodiamminedichlorido(2-(2-propynyl)octanoato)platinum(IV) [Pt(IV)Ac-POA] alone, after carbon ion irradiation (4 Gy dose) alone, or in combined exposure conditions [40 μM CDDP or 10 μM Pt(IV)Ac-POA 48-h CT + 2 or 4 Gy carbon ion radiation]. DNA counterstaining with Hoechst 33258 (*blue fluorescence*). *Scale bar*, 20 μm. Histograms (**lower right part**) represent the percentage values of caspase-3-immunopositive cells.

Based on the above-described results, we focused the initial evaluation in the REC condition on the assessment of some specific markers representative of apoptosis. Concerning caspase-3 expression (Figure 14), the samples exposed to carbon ion radiation only displayed immunopositive cell frequencies of 2.72 ± 0.60% and 4.57 ± 0.32% after the 2 and 4 Gy doses, respectively, compared to the controls (0.43 ± 0.18%); notably, the presence of cells in active mitosis was also detected, resembling the physiological condition. After 40 μM CDDP 48-h CT alone or combined with 2 or 4 Gy of carbon ion irradiation, cytotoxic effects were detected, as demonstrated by the increased immunopositive cell frequency (41.27 ± 0.57%, 44.00 ± 0.66%, and 43.08 ± 0.73%, respectively) despite the strong reduction in the cell population. After 10 μM Pt(IV)Ac-POA 48-h CT, an enhancement in caspase-3-immunopositive cell frequency was also measured (61.03 ± 0.20%), associated with a reduction in cell number. After 10 μM Pt(IV)Ac-POA 48-h CT combined with 2 or 4 Gy of carbon ion irradiation, almost all cells were caspase-3-immunopositive (92.86 ± 0.40% and 95.00 ± 0.47%, respectively), and limited cell survival was detected. Notably, these data corroborated the notion that even a low-dose carbon ion beam, i.e., 2 Gy, has a remarkable long-term effect on U251 cells. Table 7 summarizes caspase-3-immunopositive cell quantification obtained in the REC condition.

**TABLE 7:** Summary of statistical significances.

**Bonferroni’s Multiple Comparison Test**	***ρ* value**
CTR *vs.* 2 Gy *REC*	*ns*
CTR *vs.* 4 Gy *REC*	***
CTR *vs.* 40 μM CDDP *REC*	***
CTR *vs.* 10 μM Pt(IV)Ac-POA	***
CTR *vs.* 40 μM CDDP + 2Gy *REC*	***
CTR *vs.* 10 μM Pt(IV)Ac-POA + 2Gy *REC*	***
CTR *vs.* 40 μM CDDP + 4Gy *REC*	***
CTR *vs.* 10 μM Pt(IV)Ac-POA + 4Gy *REC*	***
2 Gy *REC vs.* 4 Gy *REC*	*ns*
2 Gy *REC vs.* 40 μM CDDP *REC*	***
2 Gy *REC vs.* 10 μM Pt(IV)Ac-POA	***
2 Gy *REC vs.* 40 μM CDDP + 2Gy *REC*	***
2 Gy *REC vs.* 10 μM Pt(IV)Ac-POA + 2 Gy *REC*	***
2 Gy *REC vs.* 40 μM CDDP + 4 Gy *REC*	***
2 Gy *REC vs.* 10 μM Pt(IV)Ac-POA + 4 Gy *REC*	***
4 Gy *REC vs.* 40 μM CDDP *REC*	***
4 Gy *REC vs.* 10 μM Pt(IV)Ac-POA	***
4 Gy *REC vs.* 40 μM CDDP + 2 Gy *REC*	***
4 Gy *REC vs.* 10 μM Pt(IV)Ac-POA + 2 Gy *REC*	***
4 Gy *REC vs.* 40 μM CDDP + 4 Gy *REC*	***
4 Gy *REC vs.* Pt(IV)Ac-POA + 4 Gy *REC*	***
40 μM CDDP *REC vs.* 10 μM Pt(IV)Ac-POA	***
40 μM CDDP *REC vs.* 40 μM CDDP + 2 Gy *REC*	**
40 μM CDDP *REC vs.* 10 μM Pt(IV)Ac-POA + 2 Gy *REC*	***
40 μM CDDP *REC vs.* 40 μM CDDP + 4 Gy *REC*	*ns*
40 μM CDDP *REC vs.* 10 μM Pt(IV)Ac-POA + 4Gy *REC*	***
10 μM Pt(IV)Ac-POA *REC vs.* 40 μM CDDP + 2 Gy	***
10 μM Pt(IV)Ac-POA *REC vs.* 10 μM Pt(IV)Ac-POA + 2 Gy	***
10 μM Pt(IV)Ac-POA *REC vs.* 40 μM CDDP + 4Gy	***
10 μM Pt(IV)Ac-POA *REC vs.* 10 μM Pt(IV)Ac-POA + 4 Gy	***
40 μM CDDP + 2 Gy *REC vs.* 10 μM Pt(IV)Ac-POA + 2 Gy *REC*	***
40 μM CDDP + 2 Gy *REC vs.* 40 μM CDDP + 4 Gy *REC*	*ns*
40 μM CDDP + 2 Gy *REC vs.* Pt(IV)Ac-POA + 4 Gy *REC*	***
10 μM Pt(IV)Ac-POA + 2 Gy *REC vs.* 40 μM CDDP + 4 Gy *REC*	***
10 μM Pt(IV)Ac-POA + 2 Gy *REC vs.* 10 μM Pt(IV)Ac-POA + 4 Gy *REC*	*ns*
40 μM CDDP + 4 Gy *REC vs.* 10 μM Pt(IV)Ac-POA + 4 Gy *REC*	***

Different experimental conditions (CTR, control, 40 μM CDDP and 10 μM Pt(IV)Ac-POA 48 h-CT alone or combined with 2 or 4 Gy dose irradiation); *ρ* values: ^∗^*ρ* < 0.05; ^∗∗^*ρ* < 0.01; ***ρ < 0.001, *ns*, not significant.

**FIGURE 15:**
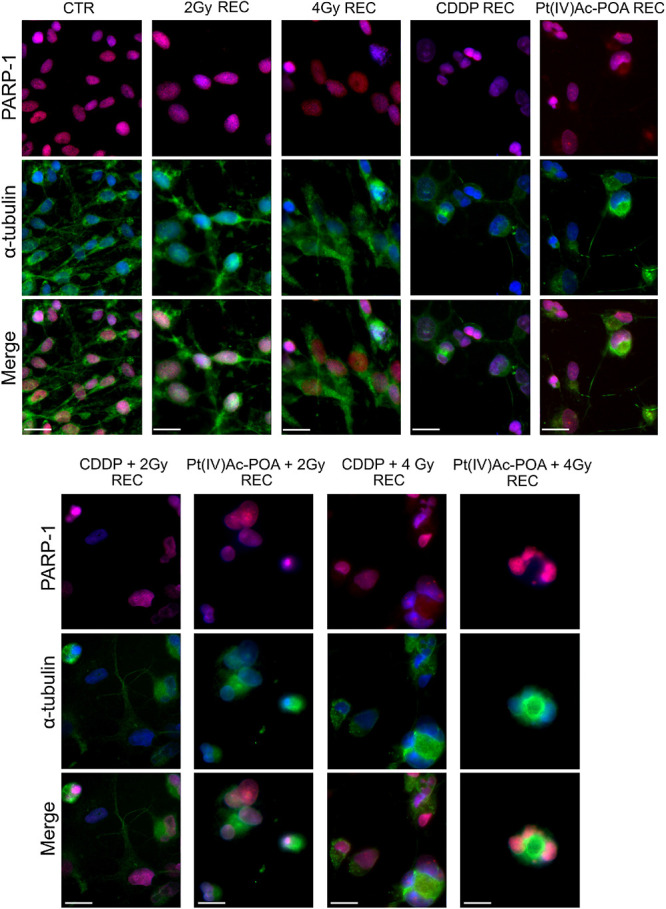
Apoptotic pathway evaluation in the recovered (REC) condition after (i) carbon ion irradiation alone or (ii) treatment with chemotherapeutics alone or (iii) combined treatments: chemotherapeutics + carbon ion radiation at different doses. Double immunofluorescence detection of PARP-1 (*red fluorescence*) and α-tubulin (*green fluorescence*) in the controls (CTR) and all different REC conditions, i.e., after 48-h CT with 40 μM *cis*-dichlorodiammineplatinum (CDDP) or 10 μM (*OC*-6-44)-acetatodiamminedichlorido(2-(2-propynyl)octanoato)platinum(IV) [Pt(IV)Ac-POA] alone, after carbon ion irradiation (2 or 4 Gy dose) alone, or in combined exposure conditions [40 μM CDDP or 10 μM Pt(IV)Ac-POA 48-h CT + 2 or 4 Gy carbon ion radiation]. DNA counterstaining with Hoechst 33258 (*blue fluorescence*). *Scale bar*, 20 μm.

With regard to PARP-1 in the REC conditions (Figure 15), any changes in immunofluorescence were detected comparing samples exposed to carbon ion radiation alone, both at 2 and 4 Gy, revealing that, at seven recovery days after irradiation, PARP-1 expression and localization at the nuclear level were comparable to the control condition. Diversely, the REC samples treated with either 40 μM CDDP or 10 μM Pt(IV)Ac-POA still demonstrated apoptosis presence, as can be deduced observing the strongly altered tubulin cytoskeleton which indicates possible cellular damage. In particular, evaluating the long-term effects after 40 μM CDDP 48-h CT followed by exposure to 2 or 4 Gy carbon ion beam, some morphological alterations were perceived, nonetheless accompanied by a spotted-like PARP-1 immunofluorescent labeling detected, supporting the occurrence of early-onset cellular recovery. Interestingly, after 10 μM Pt(IV)Ac-POA 48-h CT followed by 2 or 4 Gy carbon ion beam exposure, the translocation of PARP-1 immunofluorescence signal from the nucleus to the cytoplasm was observed, triggering apoptosis in the few surviving cells.

## Discussion

The present study was devoted to identifying a new valuable treatment useful in overcoming the limits associated with conventional cancer therapies currently used in clinical practice to treat CNS cancers. Among these limits, chemoresistance is one of the major obstacles in the treatment of nervous system (NS) tumors, as the standard protocols, as well as the treatment with TMZ, can only, in some cases, improve the patient’s prognosis, but not lead to a complete resolution. Also, the high heterogeneity that characterizes these cancers, in particular GBM, represents a great challenge for the diagnosis and the development of tumor-specific therapies.

Hence, in this view, the present investigation aimed at exploring the effects of a new platinum-based compound properly synthesized to have greater efficacy than the CDDP standard treatment, paralleled by a lower systemic toxicity. Recent data confirmed that the recently synthesized platinum-based compounds possess suitable antitumor action (Grimaldi et al., 2019; Astesana et al., 2020) and lower cytotoxic effects on healthy cells (Piccolini et al., 2015), even though the problem of acquired chemoresistance remains a topic to be[ further clarified.

In this regard, we focused on a recently lab-manufactured platinum(IV) compound, namely Pt(IV)Ac-POA, which acts as a prodrug, thus reducing the possible side effect of cytotoxicity outside tumor cells (Johnstone et al., 2016; Gabano et al., 2017). Furthermore, the ability to associate biologically active axial ligands, i.e., HDACi, within the molecule positively allows obtaining a synergistic effect that would enhance the antitumor effect.

To date, the first studies of Pt(IV)Ac-POA provided promising data in several NS tumor cell lineages (Rangone et al., 2018; Ferrari et al., 2020).

Pt(IV)Ac-POA belongs to the platinum(IV) family and performs its cytotoxic action by acting as a prodrug. Indeed, it is inactive outside the tumor cells while being activated inside them through a reduction mechanism that leads to the splitting of both the CDDP molecule and the two axial ligands including the POA (Gabano et al., 2017). Due to the presence of POA, the Pt(IV)Ac-POA molecule, as a cisplatin/POA combination molecule, increases the ability to deliver at the same time huge amounts of cisplatin and POA in cells. The “synergistic cellular accumulation” of Pt(IV)Ac-POA is mainly due to the lipophilicity of the molecule assembly with respect to the hydrophilic cisplatin and the amphiphilic POA (in anionic form at physiologic pH) precursors which allow increasing cellular uptake. Moreover, POA is considered a very active HDACi, thus indirectly producing enhanced acetylation at the chromatin level, improving decondensation and, therefore, enhancing the DNA exposure to cisplatin action (Gabano et al., 2017; Novohradsky et al., 2017), with the double consequence of inducing chemosensitization and decreasing chemoresistance. Besides, HDACi, being an epigenetic agent, affects cell fate by altering the expressions of several genes. In particular, HDACi can modulate *inter alia* the expression and function of DNA repair proteins, increasing the persistence and efficacy of the Pt-DNA adducts (Gabano et al., 2014; Kenny et al., 2017).

In the present investigation, we verified the efficacy of Pt(IV)Ac-POA on human U251 MG cell line, which possesses an extremely variable cellular phenotype, thus reflecting the typical glioblastoma high heterogeneity and invasiveness. These latter features drastically influence the efficacy of the treatments in that no specific targets have been clearly identified that could facilitate the chemotherapeutics action. Different complementary techniques have been employed to analyze firstly the effects of Pt(IV)Ac-POA administered alone and then the potential synergistic effects of treatment with the anticancer agent followed by HT with carbon ion application, with the goal to assess the potential synergistic effects of combined treatments to improve therapy efficacy.

Taking into consideration the CDDP concentration applied in previous works on different cell lines (Grimaldi et al., 2016, 2019; Astesana et al., 2020), the first experimental step was carried out to select the effective Pt(IV)Ac-POA dose. Based on the MTS viability assay data, Pt(IV)Ac-POA was demonstrated to induce cell death already at the concentration of 10 μM under the standard condition, i.e., 48-h CT, with a significant decrease (about 50%) in the number of living cells. Additionally, the clonogenic assay results provide further evidence that exposure to increasing Pt(IV)Ac-POA concentrations induced a dose-dependent impairment of U251 clonogenic ability, with the 10-μM dose able to cause a 70% colony inhibition after the chosen 10-day time window.

It has to be highlighted that, even after standard acute exposure, Pt(IV)Ac-POA was effective already at a concentration four times lower than that widely reported in *in vitro* investigations testing CDDP, describing the use of 40 μM CDDP on a variety of tumor cell lines, based on its ability to cause apoptosis (Çetin et al., 2017; Qian et al., 2018) and cell cycle and mitochondrial respiratory complex alterations (Kachadourian et al., 2007; Chiang et al., 2014). Interestingly, the data obtained by the clonogenic cell survival assay, after the 10-day time window, further demonstrated the U251 refractoriness to the CDDP treatment, after which a 50% surviving cell fraction was measured. Based on this data, it appeared clear that the CDDP-caused clonogenic inhibition is lower compared to that observed after Pt(IV)ac-POA exposure (about 70%), thus supporting a more effective long-lasting action of the prodrug on the final cell fate balance.

Cytofluorimetric results after PI staining demonstrated that Pt(IV)Ac-POA was able to induce programmed cell death through different mechanisms, even though to a lesser extent when compared with data previously observed in different cell lines (Gabano et al., 2017; Rangone et al., 2018; Ferrari et al., 2020). The presence of numerous apoptotic cells after treatment was confirmed by annexin V assay, while, at the ultrastructural level, alternative programmed cell death mechanisms, i.e., apoptosis, autophagy, and necroptosis, were identified by TEM analysis.

The encouraging aspect of 10 μM Pt(IV)Ac-POA 48-h CT was the detected lack of necrosis compared to the other concentrations used, indicating a possible lower pro-inflammatory outcome and adverse side effects for healthy cells.

Since GBM has an unfavorable prognosis mainly due to its high propensity for tumor recurrence, the “recovered condition” was used to mimic the renewal phase that follows chemotherapy treatment and therefore to observe the potential activation of resistance mechanisms in U251 cells.

Immunocytochemical staining, investigating specific representative markers, confirmed the activation of both intrinsic and extrinsic apoptotic pathways. Specifically, this technique allowed properly demonstrating the effects of 10 μM Pt(IV)Ac-POA 48-h CT and 40 μM CDDP 48-h CT, revealing that, after both treatments, cells displayed immunopositivity for both cleaved caspase-3 and caspase-8, showing an increase in the percentage of immunopositive cells compared to the control conditions. The most interesting finding was the progressive immunopositivity increase, observed in recovered cells after Pt(IV)Ac-POA exposure, showing a steady enhancement in the fluorescence signal for both caspases. This data seemed to suggest that Pt(IV)Ac-POA exposure, already at a dose of 10 μM, may have a long-lasting effect, inducing cell death over a long period after treatment. Concerning PARP-1 and RIP1, after both 10 μM Pt(IV)Ac-POA 48-h CT and 40 μM CDDP 48-h CT, an immunopositivity increase was detected. In particular, the translocation of the cleaved fragment p89 of PARP1 from the nucleus to the cell cytoplasm further confirms the activation of the intrinsic apoptotic pathway, although with a more marked effect after 10 μM Pt(IV)Ac-POA 48-h CT.

The cleavage of PARP-1 was also confirmed by the Western blotting data, by which the full length of PARP-1 and the fragment p89 were investigated. Notably, in recovered cells after 40 μM CDDP, the observed redistribution of the fluorescent PARP1 signal together with a particular *phagocytic-like* morphology may indicate a possible damage compensation played by cells, which may represent a survival mechanism after treatment.

Notably, the caspase-8 and RIP1 immunoreactivity of the Pt(IV)Ac-POA-treated cells clearly demonstrated the activation of the extrinsic apoptotic pathway, also suggesting that RIP1 translocation to the cell nucleus might represent a preliminary step of the necroptotic pathway, as observed in the treated samples by TEM.

The necroptosis triggering was also corroborated by the MLKL perinuclear localization within the fragmented nuclei in the cells treated with either CDDP or Pt(IV)Ac-POA, as previously supported by the morphological features detected by both TEM ultrastructural analysis as well as RIP1 immunofluorescence detection. This finding was particularly evident in the Pt(IV)Ac-POA REC condition, suggesting the persistence of a long-lasting activation of necroptosis after prodrug exposure. Moreover, further evidence of the involvement of different programmed cell death pathways after treatment with both platinum compounds was revealed by AIF translocation from the mitochondrial compartment to the damaged cell nuclei. This outcome, which indicated the activation of a caspase-independent pathway, was visible after both 40 μM CDDP 48-h CT and 10 μM Pt(IV)Ac-POA 48-h CT. AIF translocation was predominantly manifest after Pt(IV)Ac-POA 48-h CT and in REC samples, while it was no longer distinguishable in the CDDP REC condition. Pt(IV)Ac-POA’s ability to induce different programmed cell death pathways, lessening necrosis induction, appears to be a valuable advantage, particularly based on the observation of the enduring effect, still evident in the REC condition.

Immunofluorescence staining also demonstrated the effects of Pt(IV)Ac-POA on intracellular organelles, revealing that the prodrug is able to act on cytoplasmic targets, as previously demonstrated for other platinum(II) compounds, i.e., CDDP and Pt(*O*,*O*-acac)(γ-acac)(DMS) (Bottone et al., 2008; Santin et al., 2012; Grimaldi et al., 2016). Specifically, already at a concentration of 10 μM, Pt(IV)Ac-POA (48-h CT) affected both the mitochondria and the Golgi apparatus, which lost their physiological organization, appearing degraded and accompanied by both actin and tubulin cytoskeletal alterations.

Notably, in recovered cells after Pt(IV)Ac-POA exposure, U251 cells were characterized by an even worsened condition, showing a highly degraded Golgi cistern arrangement and a clustered mitochondria, while in recovered cells formerly treated with CDDP, a tentative cell restoration was observed, with the cytoplasmic organelles resuming their physiological structure and localization.

It has to be mentioned that mitochondrial alterations may lead to an improved ROS production and, therefore, to an increased oxidative stress status, therefore promoting better conditions to enable Pt(IV)Ac-POA activity (Muscella et al., 2011).

The present investigation also documented the autophagic pathway activation. Specifically, after 40 μM CDDP 48-h CT, the immunofluorescence result showed p62/SQSTM1-LC3B colocalization, which would be ascribable to the formation of autophagolysosomes and autophagosomes. Otherwise, after Pt(IV)Ac-POA 48-h CT, a lack of p62/SQSTM1-LC3B colocalization was detected, with the two markers displaying expression levels comparable to those observed in the controls. On the other hand, in the CDDP REC condition, an increased LC3B and p62/SQSTM1 immunoreactivity was measured, even though the two signals did not colocalize; in parallel, an enhancement in cell size was clearly observable. Differently to the effects observed after Pt(IV)Ac-POA 48-h CT, in the Pt(IV)Ac-POA REC conditions, the colocalization of p62/SQSTM1 and LC3B signals was detected. The p62/SQSTM1 expression levels measured by Western blotting analyses after 10 μM Pt(IV)Ac-POA REC supported the activation of the autophagic pathway, in which p62 may play a role.

Previous literature data demonstrated that autophagy and p62 are two interdependent parts of the protein control system, strictly interacting to maintain proteostasis, disclosing a frequent p62 upregulation and/or reduced degradation in cancer cells during tumor progression (Moscat et al., 2016; Islam et al., 2018). The p62 level homeostatic maintenance in cancers by autophagy-dependent or autophagy-independent mechanisms may contribute to the final outcome of the tumorigenic process, also having important implications for the design of forthcoming anticancer therapeutic protocols targeting autophagy or p62-regulated signaling pathways (Liu et al., 2016). The p62 levels, determined after Pt(IV)Ac-POA 48-h CT, could play a role in autophagy activation, representing a type II programmed death (Galluzzi et al., 2018) and not a cell survival mechanism (Belounis et al., 2016). This result differs from (i) the presently obtained results indicating a slight increase in p62 expression levels after both 40 μM CDDP 48-h CT as well as in recovered cells formerly exposed to CDDP, suggesting the possible occurrence of a cell survival mechanism, as also corroborated by the amelioration of the cell morphology, and (ii) previous data on cisplatin showing a chemoresistance phenomenon promoted by a strong decrease in p62 expression level (Chen et al., 2018; Lin et al., 2018).

In the second experimental phase, assessing the potential occurrence of a synergistic effect combining chemotherapeutics treatments with hadrontherapy, we firstly demonstrated that, after a 10-day time window, the clonogenicity of U251 was further decreased when exposed to combined treatments, i.e., 10 μM Pt(IV)Ac-POA + carbon ion irradiation at increasing doses (i.e., 1, 2, and 4 Gy). Specifically, a synergistic effect was detected when 10 μM Pt(IV)Ac-POA was combined with either 2 or 4 Gy radiation, with a measured reduction of colony formation of about 85%. Differently, the combined exposure to 10 μM Pt(IV)Ac-POA + 1 Gy induced a clonogenicity impairment analogous to that observed after treatment with the prodrug alone (26% *vs*. 30%). These results pertaining to the U251 clonogenicity impairment after the combined treatments provided an early evidence that exposure to a low-dose carbon ion beam, i.e., 2 Gy, was already effective to produce a long-lasting effect inducing U251 cell death.

Then, we hypothesized that the synergistic effect of chemotherapeutics plus hadrontherapy could effectively trigger and improve intrinsic apoptotic pathway activation (Di et al., 2013); therefore, caspase-3 activation and PARP-1 expression were assessed. With regard to active caspase-3, in order to compare the efficacy of the diverse combined treatments, a quantification of immunopositive cells was carried out. The measured percentages in 40 μM CDDP 48-h CT- and 10 μM Pt(IV)Ac-POA-treated cells and in the samples treated with chemotherapeutics and subsequently exposed to 2 or 4 Gy carbon ion radiation revealed a significantly increased activation of the intrinsic apoptotic pathway compared to the control condition.

Notably, the 10 μM Pt(IV)Ac-POA 48-h CT-induced cell death effect was significantly more pronounced compared to that observed after 40 μM CDDP 48-h CT exposure followed by carbon ion radiation, also highlighting that 4 Gy exposure had greater effects than the 2 Gy dose. Specifically, our data emphasize that, when preceded by Pt(IV)Ac-POA 48-h CT, the 4 Gy dose was able to induce a stronger nuclear degradation compared to the 2 Gy dose. Notably, in the recovered condition, i.e., cells exposed to 10 μM Pt(IV)Ac-POA 48-h CT followed by both 2 and 4 Gy carbon ion radiation, the efficacy of the combined treatment was particularly marked, even at the lowest dose of the high LET radiation tested.

Concerning PARP1, pivotally involved in DNA damage repair processes, it is known to act as a survival factor in case of limited damages to the DNA, while, when extensive DNA damage arises, it promotes cell death (Virág and Szabó, 2002), binding with high affinity to single- or double-strand breaking sites on the DNA (Gupte et al., 2017). Indeed, one of the immediate responses after DNA damage by ionizing radiation is the activation of poly(ADP-ribosyl)ation reaction, even if the role of PARP-1 in cluster DNA damage condition by high LET radiation is still under investigation (Atanu et al., 2016). In the present study, the localization of PARP-1 at the nuclear and cytoplasmic levels was evaluated, being the cytoplasmic translocation of cleaved PARP-1, i.e., the p89 fragment, a typical feature of apoptosis occurrence. Our findings demonstrated that the p89 fragment was detected only in selected conditions, i.e., at 40 μM CDDP 48-h CT + 4 Gy, 10 μM Pt(IV)Ac-POA 48-h CT + 2 Gy, and 10 μM Pt(IV)Ac-POA 48-h CT + 4 Gy.

It has to be recalled that, after 10 μM Pt(IV)Ac-POA 48-h CT, PARP1 was expressed at the nuclear level in cells during the early stages of apoptosis; differently, in late apoptosis, in which the nuclei were evidently fragmented, PARP1, or rather p89, moved to the cytoplasm. This latter phenomenon became particularly evident in the cells treated with 10 μM Pt(IV)Ac-POA 48-h CT and also when combining the 10 μM Pt(IV)Ac-POA 48-h CT with the following carbon ion irradiation. Taken together, the present data stressed the strongest occurrence of PARP-1 translocation in irradiated samples. Western blotting analyses disclosed a significant reduction in the expression levels of cleaved-length PARP-1 both in cells exposed to the 4 Gy irradiation alone as well as in cells exposed to 40 μM CDDP 48-h CT followed by carbon ion radiation compared to the samples treated with 10 μM Pt(IV)Ac-POA followed by 4 Gy irradiation. These findings could support the key role of PARP-1 in chemo- and radioresistance phenomena.

Surprisingly, in the samples exposed to 10 μM Pt(IV)Ac-POA followed by 4 Gy carbon ion radiation, a reduction in the expression of full-length PARP-1 paralleled by the presence of cleaved PARP-1 was detected, corroborating the strong effect of the combined 10 μM Pt(IV)Ac-POA 48-h CT + 4 Gy treatment in reducing the expression of this protein essentially involved in DNA repair.

With regard to the autophagy role, as above reported, the slight reduction in p62/SQSTM1 expression after 10 μM Pt(IV)Ac-POA 48-h CT may confirm the activation of the autophagic pathway, further validated by the Western blotting data supporting the homeostatic imbalance in the activation of autophagy. After 40 μM CDDP 48-h CT, an increased expression of p62/SQSTM1 was above described. Interestingly, this trend persisted in the samples exposed to 40 μM CDDP 48-h CT followed by 4 Gy irradiation, with a significant decrease of p62 expression indicating that the protein may be probably degraded by a strong activation of autophagy. Notably, the combined exposure to 10 μM Pt(IV)Ac-POA 48-h CT followed by 4 Gy irradiation slightly enhanced p62, which nonetheless maintained its expression value similar to that determined in the controls. According to the literature (Liu et al., 2016; Moscat et al., 2016; Islam et al., 2018), this data suggested that the p62 levels, quantitatively closer to those measured in the physiological condition, determined after the combined treatment protocol could have a role in preventing tumor progression.

When investigating the REC conditions, based on the hypothesis that the combined treatment (chemotherapeutics exposure followed by carbon ion radiation) trigger and improve apoptosis activation (Di et al., 2013), our initial experiments focused on caspase-3 activation and PARP-1 expression.

The immunofluorescence analysis for both these proteins revealed a visible restoration of the cells exposed to carbon ion radiation only, both at the 2 and 4 Gy dose. Retrieval of physiological conditions following recovery was also observed in the samples exposed to Pt(IV)Ac-POA alone. Interestingly, REC cells, formerly treated with 40 μM CDDP 48-h CT and then exposed to carbon ion radiation, both at the 2 and 4 Gy dose, displayed a reduction of caspase-3 immunopositivity, paralleled by a progressive restoration of cell morphology associated with the absence of PARP1 translocation phenomenon.

Differently, REC samples previously treated with 10 μM Pt(IV)Ac-POA 48-h CT and then irradiated at 2 or 4 Gy still showed an evident caspase-3 immunopositivity, as well as significant morphological alterations. It has to be underlined that in the Pt(IV)Ac-POA REC condition after exposure to low irradiation, i.e., 2 Gy, a significant effect, which was not already detectable in the acute condition, was perceivable in the long-term experiment. This latter data supports the hypothesis that the low-dose carbon ion beam could have better long-lasting effects compared to the 4 Gy higher dose. Taken together, these initial results suggested that the p89 fragment translocation, governed by caspase-3 activity, could be particularly effective in glioma cells irradiated with a high LET carbon ion beam (Perez et al., 2019), in which the DNA damage repair and response (DRR) to double-strand breaks (DSB) is strongly increased (Kesari et al., 2011; Erasimus et al., 2016). Lastly, these findings demonstrated that 7 days after combined treatment, 10 μM Pt(IV)Ac-POA 48-h CT + 2 or 4 Gy carbon ion beam assured the most marked effect, which has to be correlated with the chemical nature of Pt(IV)Ac-POA bearing HDACi (Oertel et al., 2011; Barazzuol et al., 2015).

In summary, our present findings support at first the use of this manufactured prodrug as a potential alternative to the use of cisplatin and its analogs, considering that Pt(IV)Ac-POA is effective in human U251 GBM cells already at a concentration four times lower than that employed for standard *in vitro* treatment with CDDP (10 *vs*. 40 μM, respectively), being able to trigger cell death pathway activation. Pt(IV)Ac-POA peculiar molecular structural stability outside cancer cells and new subcellular targets are key elements essential to counteract drug resistance also improving chemotherapeutic efficacy, thus suggesting the possibility of incorporating this prodrug into the treatment regimen for GBM. Even more, interestingly, the present investigation provides emerging evidence of the efficacy of the combined protocol using the chemotherapeutic prodrug Pt(IV)Ac-POA followed by high LET, also able to synergistically cause a long-lasting clonogenicity reduction of glioblastoma cells, thus representing a promising approach in the neoadjuvant setting to primary and recurrent glioblastomas.

## Data Availability Statement

The original contributions presented in the study are included in the article/supplementary material, further inquiries can be directed to the corresponding author/s.

## Author Contributions

MB, ER, and PR conceived and designed the experiments. BF, ER, EP, and AF performed the experiments and analyzed the data. MB, MR, FB, and CL contributed reagents, materials, and analysis tools. BF, EP, and FD searched and reviewed the literatures. BF and ER drafted the manuscript. ER, MB, AF, and PR critically revised the article. All the authors provided critical feedback, helped shape the research and analysis, read and agreed to the published version of the manuscript.

## Conflict of Interest

The authors declare that the research was conducted in the absence of any commercial or financial relationships that could be construed as a potential conflict of interest.
